# The Antiviral Activity of Bacterial, Fungal, and Algal Polysaccharides as Bioactive Ingredients: Potential Uses for Enhancing Immune Systems and Preventing Viruses

**DOI:** 10.3389/fnut.2021.772033

**Published:** 2021-11-05

**Authors:** Worraprat Chaisuwan, Yuthana Phimolsiripol, Thanongsak Chaiyaso, Charin Techapun, Noppol Leksawasdi, Kittisak Jantanasakulwong, Pornchai Rachtanapun, Sutee Wangtueai, Sarana Rose Sommano, SangGuan You, Joe M. Regenstein, Francisco J. Barba, Phisit Seesuriyachan

**Affiliations:** ^1^Interdisciplinary Program in Biotechnology, Graduate School, Chiang Mai University, Chiang Mai, Thailand; ^2^Faculty of Agro-Industry, Chiang Mai University, Chiang Mai, Thailand; ^3^Cluster of Agro Bio-Circular-Green Industry (Agro-BCG), Chiang Mai University, Chiang Mai, Thailand; ^4^College of Maritime Studies and Management, Chiang Mai University, Samut Sakhon, Thailand; ^5^Plant Bioactive Compound Laboratory (BAC), Department of Plant and Soil Sciences, Faculty of Agriculture, Chiang Mai University, Chiang Mai, Thailand; ^6^Department of Marine Food Science and Technology, Gangneung-Wonju National University, Gangneung, South Korea; ^7^Department of Food Science, College of Agriculture and Life Science, Cornell University, Ithaca, NY, United States; ^8^Department of Preventive Medicine and Public Health, Food Science, Toxicology and Forensic Medicine, Faculty of Pharmacy, Universitat de València, Valencia, Spain

**Keywords:** sulfated polysaccharides, immunomodulation, SARS-CoV-2, COVID-19, antiviral activity

## Abstract

Viral infections may cause serious human diseases. For instance, the recent appearance of the novel virus, SARS-CoV-2, causing COVID-19, has spread globally and is a serious public health concern. The consumption of healthy, proper, functional, and nutrient-rich foods has an important role in enhancing an individual's immune system and preventing viral infections. Several polysaccharides from natural sources such as algae, bacteria, and fungi have been considered as generally recognized as safe (GRAS) by the US Food and Drug Administration. They are safe, low-toxicity, biodegradable, and have biological activities. In this review, the bioactive polysaccharides derived from various microorganisms, including bacteria, fungi, and algae were evaluated. Antiviral mechanisms of these polysaccharides were discussed. Finally, the potential use of microbial and algal polysaccharides as an antiviral and immune boosting strategy was addressed. The microbial polysaccharides exhibited several bioactivities, including antioxidant, anti-inflammatory, antimicrobial, antitumor, and immunomodulatory activities. Some microbes are able to produce sulfated polysaccharides, which are well-known to exert a board spectrum of biological activities, especially antiviral properties. Microbial polysaccharide can inhibit various viruses using different mechanisms. Furthermore, these microbial polysaccharides are also able to modulate immune responses to prevent and/or inhibit virus infections. There are many molecular factors influencing their bioactivities, e.g., functional groups, conformations, compositions, and molecular weight. At this stage of development, microbial polysaccharides will be used as adjuvants, nutrient supplements, and for drug delivery to prevent several virus infections, especially SARS-CoV-2 infection.

## Introduction

Viruses are the most numerous living organisms on the earth and can be found in terrestrial and aquatic environments. They are infectious agents containing a genetic material within a protein coat—requiring an appropriate host cell where they can replicate (called infection) often resulting in diseases. Viruses can infect all types of organisms: prokaryotes (archaea and bacteria), eukaryotes (animals, algae, plants, and protozoa), and giant viruses namely virophages (1). Like other viruses, human viruses are able to replicate and mutate. A new virus was discovered in December 2019 and characterized as a pandemic by World Health Organization (WHO) on March 11, 2020 (2). This virus was characterized as the severe acute respiratory syndrome coronavirus 2 (SARS-CoV-2), which causes human infection called coronavirus disease 2019 (COVID-19). The disease has spread worldwide and caused over 200 million cases and 4 million deaths from its start until August 2021 (3).

Coronaviruses (CoV) are enveloped positive-sense single-stranded RNA (+ ssRNA) viruses with crown-like spikes on their spherical surface (4). Coronaviruses belong to the order *Nidovirales*, the suborder *Coronavirineae*, and the family *Caronaviridae*. The family was divided into the subfamilies of *Orthocoronavirinae* and *Letovirinae* by the International Committee on Taxonomy of Viruses (ICTV) in 2018 (5). The former sorts into 4 genera, including *Alphacoronavirus, Betacoronavirus, Gammacoronavirus*, and *Deltacoronavirus*, with α- and β-coronaviruses infect mammalian species, but γ-, and δ-coronaviruses infect avian species causing respiratory and enteric diseases both as acute and persistent infections (6, 7). The human coronavirus first emerged in patients with the common cold in the 1960s. Other human coronaviruses have emerged within the last two decades: SARS-CoV-1 (2003), Middle East respiratory syndrome (MERS-CoV, 2012), as well as SARS-CoV-2 (2019), which is the seventh human-infecting coronavirus identified (7).

**Figure 1:**
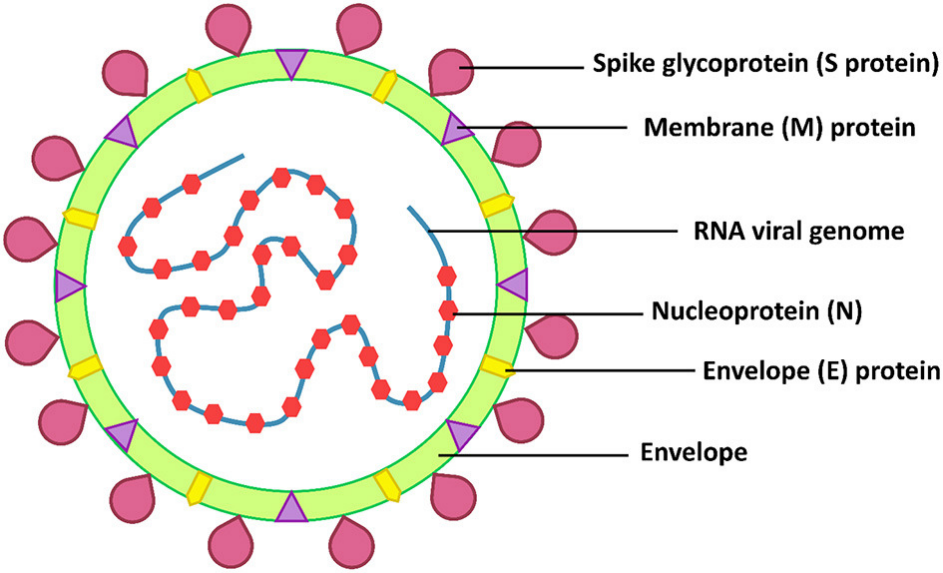
SARS-CoV-2 structure.

SARS-CoV-2 belongs to the β-coronaviruses, which include SARS-CoV-1 and MERS-CoV. These viruses are highly pathogenic and have a high mortality rate (8). The RNA genome of SARS-CoV-2 is 25–32 kb and similar to SARS-CoV-1 (82% similarity) (4). The structural proteins of SARS-CoV-2 are the envelope (E), membrane (M), nucleocapsid (N), and spike (S) proteins (Figure 1). The spike of coronaviruses (S protein) is a glycoprotein associated with the pathogenesis because it is involved in virus adsorption and entry. Thus, the S protein is the virus' important virulence factor (5). The virus uses the S protein for cell binding and membrane fusion. The S protein binds to the angiotensin-converting enzyme 2 (ACE2), a host cell receptor, primed by the transmembrane protease serine protease 2 (TMPRSS2), with the interaction mediating the virus attachment and entry into a host cell (5, 6, 9). The ACE2 receptor is found in a range of human tissues and organs, including the small intestine, lungs, heart, testis, kidneys, blood vessels, muscle, adipose tissues, bladder, epithelia cells of the oral cavity, and the upper esophagus (10–12). Therefore, there are many targets for SARS-CoV-2 infection.

Four antiviral drugs were repurposed for COVID-19, including tocilizumab, remdesivir, favipiravir, and dexamethasone (13). Remdesivir, a drug approved by the US Food and Drug Administration (FDA) for COVID-19, showed a high efficacy as a COVID-19 treatment (14, 15). On the other hand, the WHO Solidarity Trial Consortium announced that remdesivir had little or no effect on hospitalized patients with COVID-19 (16). Various researchers have been evaluating and developing other antiviral agents, as well as vaccines, with high efficacy and low-toxicity (17). As of August 2021, people have been administered different Covid vaccines in many countries, but the pandemic still goes on. Nutrients in foods have an important role in stimulating human immunity and preventing viral infections. Several nutrients including polysaccharides, proteins, and lipids have been reported to have antiviral and immune-enhancing properties. In addition, micronutrients, such as vitamin A, C, D, and E, and few minerals, such as iron, selenium, and zinc have the potential to improve the immune system. Furthermore, natural extracts containing some non-nutrients, such as polyphenols, flavonoids, alkaloids, thiophenes, terpenoids, tannins, and lignins have also shown biological activities (18). For instance, *Erodium glaucophyllum* extracts containing phenolic compounds such as gallic acid, quercetin 3-O-glucuronide, (+)-gallocatechin, and (+)-catechin exhibited antibacterial and antiviral activities (19). During the COVID-19 pandemic, healthy, nutrient-rich, and functional foods may be more important because their consumption may prevent and modulate the immune system (20). In addition, the development of bioactive ingredients, functional, and nutrient-rich foods that can moderate consumers' overall health will be more interested (21).

Polysaccharides are polymeric carbohydrates, defined as composed of more than 10 monomers that are linked by glycosidic linkages. Polysaccharides are grouped into 2 classes: homopolysaccharides (contain one type of monomer) and heteropolysaccharide (contain more than one type of monomer) (22). Polysaccharides from each sources have different branched chains, composition of monosaccharides, molecular weight (MW), and structural conformations (23). Polysaccharides are the most abundant biological macromolecules in nature and can be obtained from every living organisms including animals (24), plants (25), and microorganisms (26). In living cells, polysaccharides are involved in structure, storage, adhesion, and cell recognition (27). Microorganisms including archaea, bacteria, fungi, and microalgae produced diverse polysaccharides with different structures and functions. Moreover, microorganisms synthesize polysaccharides and secrete them to the outside, these are called exopolysaccharides (EPS). Their functions include cell adhesion, migration of bacteria in groundwater, protection from predators and white blood cells, protection from undesired environments (extreme environments), intercellular signal transduction, and molecular recognition (28, 29). Microbial polysaccharides are composed of not only monosaccharides, but also proteins, lipids, metal ions, extracellular DNA (eDNA), and other organic and inorganic compounds (30). Furthermore, polysaccharides derived from microorganisms, especially marine microorganisms may include sulfate groups, and are called sulfated polysaccharides (31). Sulfated polysaccharides are negatively-charged biopolymers found in the cell wall of marine algae (green, brown, and red algae). Sulfate groups are linked to the sugar structure's backbone to stabilize the structure in extreme environments, especially high salinity (32). Sulfated polysaccharides can be founded not only in marine microalgae and macroalgae, but also in marine animals, and marine bacteria (31). Microbial polysaccharides and sulfated polysaccharides show various biological activities such as immunomodulatory, antioxidant, antimicrobial, anticancer, and anti-inflammatory activities (30). In particular, the antiviral activity of microbial polysaccharides has been studied, showing in several cases an inhibitory effect against various animal, human, and plant pathogenic viruses (33–35). Many studies have reported that natural and modified polysaccharides could inhibit various virus infections (36). Some microbial polysaccharides had antiviral activity against various viruses including *Herpes simplex*, influenza, Newcastle disease (NDV), *Varicella zoster* (VZV), human immunodeficiency viruses (HIV), and human adenoviruses (37–45). According to their biological activities, the bioactive polysaccharides can be applied as a bioactive ingredients to improve the immune system and reduce the damage caused by viruses (23). Among microbial polysaccharides, EPS produced by lactic acid bacteria (LAB) have been recognized as GRAS, which allows their use in food without the need for regulatory oversight in the USA (46). Although bioactive polysaccharides have been derived from plants, many researchers have investigated the characteristics, compositions, properties, biological activities of novel polysaccharides from various microorganisms (30). There are many advantages to using microbial polysaccharides compared to other polysaccharides. For example, the microbial polysaccharide production can be done using optimized conditions indoors. Microbes grow easily and fast with a high yield of polysaccharides. The recovery process of polysaccharides is simple. Moreover, microbial growth media are simple and non-toxic. If agricultural wastes are used as microbial growth media, the cost of the production is often decreased (32, 47). Microbial polysaccharides are biocompatible and biodegradable, and have no known toxic effects (23). As mentioned above, microbial polysaccharides show antioxidant, anti-inflammatory, antiviral, and immunomodulatory activities; therefore, microbial polysaccharides are attractive as antiviral agents or bioactive ingredients to treat viral infectious diseases, especially COVID-19. This review focuses on microbial polysaccharides with antiviral and immunomodulatory activities and their antiviral mechanisms, and provides the potential approach to use microbial polysaccharides as bioactive ingredients.

## Microbial Polysaccharides With Antiviral Activity

### Antiviral Polysaccharides From Algae

Algae are eukaryotic photosynthetic organisms, often microorganisms. Some algae are unicellular, but some of them are multicellular organisms lacking of specialized tissues. Both micro- and macroalgae are good sources of biomedical compounds, especially polysaccharides (48). Algal polysaccharides are nontoxic, edible, biocompatible, biodegradable, and easily available; therefore, these biopolymers have been applied in many fields such as the food, pharmaceutical, and biomedical industries (49). Algal polysaccharides have several pharmaceutical properties, including anticancer (50), antioxidant (51), antimicrobial (52), anti-inflammatory (53), and immunomodulatory activities (54). Moreover, several algae, especially marine algae, can produced sulfate polysaccharides, which have different beneficial biological activities (50, 55–58). Different algal polysaccharides possess a variety of structures, composition, and conformations, which influence their properties (55). A summary of algal polysaccharides with antiviral potential are shown in Table 1.

**Table 1:** The microbial polysaccharides with antiviral activity.

**Source**	**Polysaccharide**	**Virus**	**Action**	**References**
**Algae**				
*Coccomyxa gloeobotrydiformi*	Acidic polysaccharide (CmAPS)	Human influenza A virus: A/H1N1, A/H2N2, A/H3N2 and A/H1N1	Inhibited virus adsorption and virus-induced erythrocyte hemagglutination and hemolysis	(40)
*Gracilaria lemaneiformis*	Sulfated polysaccharide	Human influenza virus H1-364	Prevented virus adsorption and replication	(59)
*Gyrodinium impudicum*	Sulfated exopolysaccharide (p-KG03)	Ecephalomyocarditis virus (EMCV)	Inhibited EMCV infection in HeLa cells	(56)
*Gyrodinium impudicum*	Sulfated exopolysaccharide (p-KG03)	Influenza A virus	Inhibition of influenza virus replication	(60)
*Himanthalia elongata*	Polysaccharide	HSV-1	N/A	(61)
*Hydroclathrus clathratus*	Sulfated polysaccharide HC-b1	HSV-1, including acyclovir-resistant strain and clinical strain	Inhibited virus absorption and penetration, and inhibited replication of HSV-1 in host cells	(62)
*Hydroclathrus clathratus*	Sulfated polysaccharide	HSV-2	N/A	(63)
*Laminaria japonica*	Polysaccharide	Respiratory syncytial virus (RSV)	Inhibited RSV replication and induced IFN-α secretion	(64)
*Laminaria japonica*	LJ04 polysaccharide	Enterovirus 71 (EV71)	Inhibited viral proliferation, viral-induced apoptosis, and increased IFN-β expression	(65)
*Laminaria japonica*	Fucoidan	I-type influenza virus, adenovirus and parainfluenza virus I	N/A	(66)
*Navicula directa*	Naviculan	HSV-1, HSV-2, and Influenza A virus (H1N1)	Inhibited viral adsorption and penetration	(67)
*Padina tetrastromatica*	Polysaccharide	Herpes simplex virus type 1 and 2 (HSV-1 and HSV-2)	Inhibited virus adsorption	(37)
*Porphyridium cruentum*	i Polysaccharide	Varicella zoster virus (VZV)	i Polysaccharide may block different phases of viral replication cycle	(43)
*Porphyridium* sp.	Cell-wall sulfated polysaccharide	HSV-1, HSV-2, and VZV	Inhibited virus adsorption and/or production of new virions in host cells	(38)
*Porphyridium* sp., *P. aerugineum, and Rhodella reticulata*,	Polysaccharide	Murine leukemia virus (MuLV) and murine sarcoma virus (MuSV-124)	Inhibition of virus adsorption	(68)
*Saccharina japonica*	Sulfated galactofucan (SJ-D-S-H) and glucuronomannan (Gn)	SARS-CoV-2	Binding SARS-Cov-2 spike glycoprotein	(69)
*Sargassum fusiforme*	Polysaccharide SFP	Avian leukosis virus subgroup J (ALV-J)	Inhibited on virus adsorption phase by binding to virions and showed inhibitory effects both *in vitro* and *in vivo*	(70)
*Sargassum patens*	Sulfated polysaccharide SP-2a	HSV-1	Inhibition of virus adsorption	(71)
*Sargassum trichophyllum*	Fucoidan	HSV-2	Inhibition of virus adsorption and/or virus penetration steps	(72)
*Ulva lactuca*	Sulfated polysaccharide	Japanese encephalitis virus (JEV)	Inhibited virus adsorption	(73)
**Bacteria**				
*Arthrospira platensis*	Spirulan-like molecules	Human cytomegalovirus, HSV-1, human herpesvirus type 6 and HIV-1	Inhibited the herpesviruses at an entry phase, but at a stage later than virus entry for HIV	(44)
*Arthrospira platensis*	Calcium spirulan (Ca-SP)	HSV-1, human cytomegalovirus, measles virus, mumps virus, influenza A virus, and HIV-1	Inhibited the penetration of virus into host cells	(74)
*Arthrospira platensis*	Exopolysaccharide	Koi herpesvirus (KHV)	Inhibited the viral replication	(75)
*Bacillus licheniformis* T14	Exopolysaccharide	HSV-2	Inhibited virus replication in human peripheral blood mononuclear cells (PBMC)	(76)
*Lactobacillus delbrueckii* OLL1073R-1	Exopolysaccharide	Intestinal viruses	Increased the expression of the antiviral factors MxA and RNase L	(77)
*Lactobacillus plantarum* strain N4(Lp)	Exopolysaccharide	Transmissible Gastroenteritis Virus (TGEV) - Coronavirus	Inhibition effect that co-incubation with TGEV “Coronavirus”	(78)
*Lactobacillus* spp.	Exopolysaccharide 26a	Human adenovirus type 5 (HAdV-5)	Suppressed the formation and release of HAdV-5 virions	(41)
*Nostoc flagelliforme*	Nostoflan	HSV-1, HSV-2,HCMV, and influenza A virus	Blocked virus adsorption and/or virus penetration steps	(79)
*Pseudomonas* sp. WAK-1	Extracellular glycosaminoglycan and sulfated polysaccharide	HSV-1, Influenza A virus	N/A	(80)
**Fungi**				
*Auricularia auricula*	Sulfated *Auricularia auricula* polysaccharide (AAPt)	Newcastle disease virus	Inhibit the cellular infectivity (in chicken embryo fibroblast, CEF) of NDV in three ways (pre-, post- and simultaneous-adding polysaccharide)	(42)
*Fomes fomentarius*	Polysaccharide BAS-F	Tobacco mosaic virus (TMV)	N/A	(34)
*Inonotous obiquus*	*Inonotus obliquus* polysaccharides (IOPs)	Feline calicivirus (FCV) strain F9, feline herpesvirus 1, feline influenza virus H3N2 and H5N6, feline panleukopenia virus and feline infectious peritonitis virus	Antiviral effects on virus particles through blocking viral binding/absorption	(35)
*Porodaedalea pini* (Brot.) Murrill (syn. *Phellinus pini*)	Polysaccharide EP-AV1 and EP-AV2	HSV-1, coxsackie virus B3 (CVB3)	N/A	(81)
*Grifola frondosa*	Heteropolysaccharide GFP1	Enterovirus 71	Inhibited EV71 replication and suppressed viral VP1 protein expression and genomic RNA synthesis	(82)
*Lentinus edodes*	Lentinan	Infectious hematopoietic necrosis virus (IHNV)	Inhibited viral replication	(83)
*Cordyceps militaris*	Acidic polysaccharide (APS)	Influenza A virus	Reduced virus titer the bronchoalveolar lavage fluid and the lung of mice infected with influenza A virus	(39)

*N/A, Not available*.

Most of the algal polysaccharides have the ability to decrease viral infections by blocking the attachment of virus particles to host cell surfaces. In this line, three polysaccharides extracted from *Sargassum trichophyllum* (a brown alga) were characterized as laminaran, alginate and fucoidan, observing that only fucoidan showed an antiviral effect against herpes simplex virus type 2 (HSV-2) (72). The 50% inhibitory concentration (IC_50_) of the fucoidan was 18 μg·mL^−1^ when it was added during the viral infection, being lower than adding after the viral infection. Therefore, this polysaccharide might inhibit HSV-2 at the virus adsorption and/or penetration step(s) (72). *Sargassum henslowianum* produced antiviral fucoidans against both HSV-1 and HSV-2 (84). For instance, the authors observed how two fractions of the fucoidans (SHAP-1 and SHAP-2) could inhibit HSV-1 with IC_50_ of 0.89 and 0.82 μg·mL^−1^, respectively. Both SHAP-1 and SHAP-2 showed higher antiviral activity against HSV-2 with IC_50_ of 0.48 μg·mL^−1^. These fucoidans interfered with the virions' attachment to host cells (84). Moreover, low MW fucoidan fractions (LF1 and LF2) from *Laminaria japonica* could inhibit I-type influenza virus, adenovirus and parainfluenza virus I *in vitro*. The IC_50_ for LF1 were 0.3, 0.6, and 0.3 mg·mL^−1^, respectively, whereas The IC_50_ for LF2 were 0.6, 1.2, and 0.6 mg·mL^−1^, respectively (66). Fucoidan from *Cladosiphon okamuranus* also showed higher antiviral activity against NDV with lower cytotoxicity than Ribavirin, an antiviral drug, preventing this polysaccharide the viral infection at early steps by blocking the F protein (85). In addition, *Scytosiphon lomentaria*, a brown seaweed, also produced fucoidans with antiviral activity, in particular, they had the ability to block HSV-1 and HSV-2 infections (86). Moreover, a fucoidan with high levels of sulfate groups also showed the highest antiviral activity against HSV-1 and HSV-2 (86).

Carrageenans are sulfated linear polysaccharides extracted from some red algae, such as *Chondrus, Gigartina, Hypnea*, and *Eucheuma* spp. (49). These polysaccharides showed an antiviral activity against several viruses. For instance, González et al. (87) reported a good inhibitory effect of carrageenan against some enveloped viruses, including HSV-1, HSV-2, Semliki Forest, vaccinia, and swine fever viruses, but they did not find any impact on vesicular stomatitis and measles viruses. Moreover, these authors also showed the antiviral activity of carrageenan against encephalomyocarditis virus (EMCV), a naked virus, but they did not observe significant effects on poliovirus or adenovirus. The carrageenan interfered with the viral protein synthesis. On the other hand, λ-carrageenan and moderately cyclized μ/ℓ-carrageenan extracted from a red seaweed (*Gigartina skottsbergii*) inhibited the viral attachment of HSV-1 and HSV-2 (88). Various types of carrageenans also have shown antiviral activities against hepatitis A virus (HAV). The 50% effective dose (ED_50_) for ι-carrageenan, λ-carrageenan, and κ-carrageenan against HAV were >400, >222, and >10 μg·mL^−1^, respectively (89). λ-Carrageenan from *G. skottsbergii* showed an inhibitory effect on both bovine herpesvirus type 1 (BoHV-1) and Suid herpesvirus type 1 (SuHV-1). The IC_50_ of this polysaccharide was 0.52 and 10.4 μg·mL^−1^, respectively (90).

The red micro algae, *Porphyridium* spp., produce antiviral polysaccharides against many types of viruses, including HSV-1, HSV-2, and VZV. The algae inhibited viral entry and/or blocked virus replication in host cells (38, 43). In this line, the sulfated polysaccharide SP-2a obtained from a brown alga, *Sargassum patens*, exhibited strong antiviral property against different strains of HSV-1. The EC_50_ of SP-2a against the standard, acyclovir (ACV)-sensitive and -resistant strains of HSV-1 were 5.5, 1.5, and 4.1 μg·mL^−1^, respectively. The SP-2a had a weak virucidal activity against the standard and ACV-sensitive strains of HSV-1, but not the ACV-resistant strain. During virus adsorption, the SP-2a showed ≥80% inhibition of adsorption against all strains of HSV-1 (71). p-KG03 is a sulfated exopolysaccharide with an average MW of 1.87 × 10^7^ Da extracted from a dinoflagellate, *Gyrodinium impudicum* strain KG03. The p-KG03 could inhibit EMCV in HeLa cells with an EC_50_ of 26.9 μg·mL^−1^, and influenza A at the virus adsorption step, but was ineffective against influenza B, HSV-1, HSV-2, human immunodeficiency virus type 1 (HIV-1), HIV-2, Coxsackie B virus type 3 (Cox-B3), and vesicular stomatitis virus (VSV). In addition, the p-KG03 also showed antiviral activity against influenza A virus at the virus adsorption step, but did not inhibit all influenza B virus isolates. The EC_50_ for p-KG03 against different strains of influenza A virus (H1N1: PR8 and Tw; H3N2: Se) ranged from 0.19 to 0.48 μg·mL^−1^ (56, 60). Lee et al. (67) reported that *Navicula directa*, a diatom collected from deep-sea water in Toyama Bay (Japan), produced naviculan (a sulfated polysaccharide). The naviculan is a heteropolysaccharide consisting of fucose, xylose, galactose, mannose, rhamnose, and sulfate with an average MW of ~2.2 × 10^5^ Da. It is a broad-spectrum antiviral against HSV-1, HSV-2, and influenza A virus with IC_50_ of 14, 7.4, and 170 μg·mL^−1^, respectively, at the virus adsorption phase. Moreover, it could also interfere with the cell-cell fusion of HIV gp160- and CD4-expressing HeLa cells. Therefore, it might prevent HIV infections.

### Antiviral Polysaccharides From Bacteria

Bacteria (including cyanobacteria or blue-green algae) have the ability to synthesize polysaccharides for various purposes such as storage, cell protection, and adhesion. Polysaccharides accumulated in cells are called intracellular polysaccharides (ICP). While those outside of cell are called extracellular polysaccharides or EPS. The latter are secreted by cells or produced extracellularly using cell wall-anchored enzymes (28). Bacterial polysaccharides show biological (bioactive) activities, including anti-inflammatory, anticancer, antimicrobial, antioxidant, and immunomodulatory (91–97). They showed an inhibitory effect against various viruses, both DNA and RNA viruses. The inhibitory effect is usually associated with the viral adsorption and/or replication phases in host cells. For example, *Arthrospira platensis* (formerly *Spirulina platensis*) produced calcium spirulan, a sulfated polysaccharide, with antiviral activity against several enveloped viruses. The calcium spirulan composed of rhamnose, ribose, mannose, fructose, galactose, xylose, glucose, glucuronic acid, galacturonic acid, sulfate, and calcium. This polysaccharide showed antiviral activity against HSV-1, human cytomegalovirus (HCMV), measles, mumps, influenza A, and HIV-1 viruses by inhibiting virus penetration (74). Spirulan-like substances extracted from *A. platensis* showed strong antiviral activity against HCMV, HSV-1, human herpesvirus type 6 (HHV-6), and HIV-1. Their mechanisms depended on the type of virus. For example, in the case of herpesviruses, spirulan-like substances inhibited virus adsorption and/or penetration steps, while HIV-1 was inhibited after viral entry. For HCMV, the inhibition occurred at intracellular steps, especially the viral protein synthesis step (44). EPS from *A. platensis* also inhibited koi herpesviruses (KHV). Reichert et al. (75) reported that EPS from *A. platensis* between 18 and 36 μg·mL^−1^ suppressed KHV *in vitro* Furthermore, nostoflan, an acidic polysaccharide from *Nostoc flagelliforme* (a cyanobacterium), showed an interesting antiviral activity against enveloped viruses: HSV-1, HSV-2, HCMV, and influenza A viruses. The virus infections were blocked when nostoflan was added at the same time as viral infections. Therefore, nostoflan blocked the viruses at the virus adsorption stage. The IC_50_ values of nostoflan for HSV-1, HSV-2, HCMV, and influenza A viruses were 0.37, 2.9, 0.47, and 78 μg·mL^−1^, respectively (79). In addition, other authors also observed how an EPS derived from *Bacillus licheniformis* strain T14 can prevent HSV-2 infection at 300 and 400 μg·mL^−1^ in human peripheral blood mononuclear cells (PBMC) (76). EPS26a from *Lactobacillus* sp. could completely inhibit human adenovirus type 5 (HAdV-5) formation and release (41).

Bacterial polysaccharides also indirectly inhibited virus infections by modulation of the immune response. For instance, an EPS produced by *Lactobacillus delbrueckii* OLL1073R-1 activated the Toll-like receptor 3 (TLR3) and the expression of interferon (IFN)-α, IFN-β, MxA, and RNase L in porcine intestinal epithelial (PIE) cells, which was associated with the innate antiviral immune response (77). Mizuno et al. (98) reported that an EPS from *Streptococcus thermophilus* ST538 can activate TLR3, thus promoting the expression of IFN-β, interleukin 6 (IL-6), and C-X-C motif chemokine 10 (CXCL10). Antiviral bacterial polysaccharides are also shown in Table 1.

### Antiviral Polysaccharides From Fungi

Fungi are unicellular-to-multicellular eukaryotic microorganisms. They can produce a plethora of biologically active compounds, especially secondary metabolites. Similar to algae and bacteria, fungal polysaccharides (primary metabolites) also showed antiviral activity. Fungal polysaccharides can be derived from culture broth, mycelial culture and/or fruiting bodies (99). Fungal polysaccharides, such as glucan, chitin, mannan, PSK or lentinan, showed antiviral potential against animal, human, and plant viruses (100–103). Fungal polysaccharides with antiviral activity are summarized in Table 1. For example, BAS-F, a polysaccharide from *Fomes fomentarius*, at 2 μg·mL^−1^ can prevent tobacco mosaic virus (TMV) infection on the leaf surfaces (34). *Porodaedalea pini* (formerly known as *Phellinus pini*) produced two antiviral polysaccharides (EP-AV1 and EP-AV2) against HSV-1 and coxsackie virus B3 (CVB3) in Vero and HeLa cells, respectively. The EP-AV2 with a lower MW (~100 kDa) showed more potent antiviral activity than EP-AV1 (~1,010 kDa) against CVB3. It was observed how EP-AV1 and EP-AV2 polysaccharides inhibited the plaque formation caused by CVB3 in HeLa cells by 32 and 84% at 1 mg·mL^−1^, respectively. These polysaccharides specifically inhibited HSV-1 more than CVB3 as indicated by their EC_50_ values. The EC_50_ values of EP-AV1 and EP-AV2 for HSV-1 were 0.20 and 0.21 μg·mL^−1^, respectively, whereas CVB3 were 1 and 0.576 mg·mL^−1^, respectively (81). Furthermore, a polysaccharide extracted from the mycelium and fruiting body of *Lentides edodes* was able to inhibit poliovirus type 1 (PV-1) and bovine herpes virus type 1 (BoHV-1) with IC_50_ values of 0.19 and 0.1 mg·mL^−1^, respectively (101). In another study, *Grifola frondosa* mycelia were evaluated as a source of antiviral polysaccharides, observing that it had the antiviral polysaccharide, GFP1. This polysaccharide was a heteropolysaccharide containing glucose and fucose with a MW of ~40.5 kDa. Zhao et al. (82) reported that GFP1 blocked enterovirus 71 (EV71) infection at the virus replication phase. The GFP1 suppressed the viral protein expression and viral RNA genome synthesis. Fungal polysaccharides also showed important antiviral properties against animal viruses. For example, a polysaccharide from *L. edodes*, called lentinan comprised of glucose, mannose, and galactose with MW of ~3.79 × 10^5^ Da showed antiviral activity against infectious hematopoietic necrosis virus (IHNV) infecting rainbow trout (*Oncorhynchus mykiss*) and several species of salmon. The LNT-I acted both direct inactivation and inhibition of viral replication with 62.3, and 82.4% inhibition, respectively (83). *Inonotus obliquus*, chaga mushroom, also produced broad-spectrum antiviral polysaccharides against feline viruses. The polysaccharides suppressed infections of feline calicivirus (FCV), feline herpesvirus 1 (FHV-1), feline panleukopenia (FPV), feline coronavirus (FCoV), and feline influenza (FIV, H3N2, and H5N6) viruses. These polysaccharides had low toxicity and blocked virus entry by affecting the virions and/or the receptor(s) on host cell surfaces (35).

Therefore, polysaccharides from different species show various biological activities with different levels of action. Sulfated polysaccharides derived from marine microalgae and seaweeds showed many different bioactive properties and were effective against viruses at low concentrations, compared to other polysaccharides. In addition, bacteria and fungi are easily grow on simple media or agricultural wastes. The production can be done using controllable conditions and they produce high amounts of polysaccharides. Therefore, it would also benificial if the bioactivities and physicochemical properties of bacterial and fungal polysaccharides could be modified. The molecular modification of polysaccharides is an alternative approach to modulate their properties.

## Antiviral Mechanisms of Microbial Polysaccharides

In viral replication, there are 6 major steps during the infection: (1) virus attachment, (2) penetration, (3) uncoating, (4) genome replication and protein synthesis, (5) viral assembly, and (6) release of new virions (104). Different microbial polysaccharides have different antiviral mechanisms depending on virus types. The polysaccharides mostly prevented the initial steps of the virus life cycle. They interacted with virus particles and/or receptors on host cells to interfere with virus adsorption and invasion. However, some microbial polysaccharides could inhibit viral replication and protein translation. While others showed immune-enhancing activity, especially antiviral immune responses, which prevent virus infections and reduce disease severity (31, 105).

### Inactivating Virus Particles Directly

**Figure 2:**
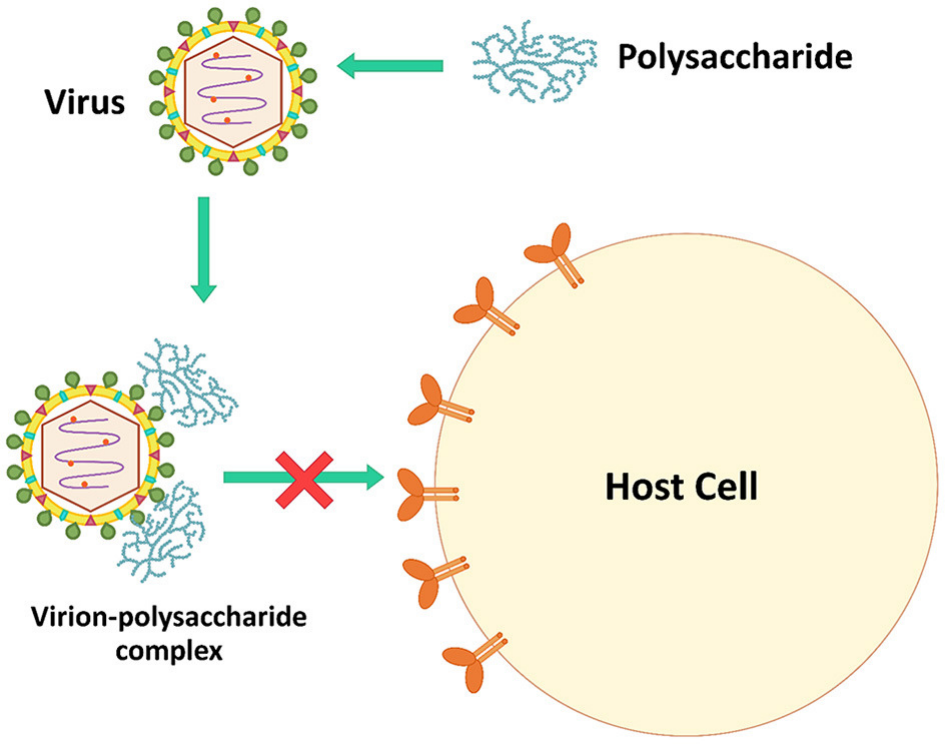
The inhibition mechanism by directly inactivating virus.

Microbial polysaccharides, especially sulfated polysaccharides, have a negative charge that can interact directly with the viral surfaces. The virucidal activity of microbial polysaccharides is caused by theses interactions (106). The complexes interfere with the viral infection process, reducing viral proliferation in host cells (Figure 2). For example, polysaccharides extracted from *Auricularia auricular*, a basidiomycete mushroom, can inhibit NDV in CEF cells. During the process of adding polysaccharides and virus simultaneously, the virus inhibitory rates were higher than pre- and post-addition of the polysaccharides. These polysaccharides might be combined with virus particles to block virus attachment to host cells (42). *Inonotus obliquus* polysaccharides also directly blocked feline virus virions (FCV, FHV-1, FPV, feline coronavirus FCoV, and FIV). These polysaccharides were mixed with the viruses for 1 h before adding to the cell lines, decreasing significantly the viral infectivity compared to untreated viruses (35).

### Inhibiting Virus Adsorption and Penetration

**Figure 3:**
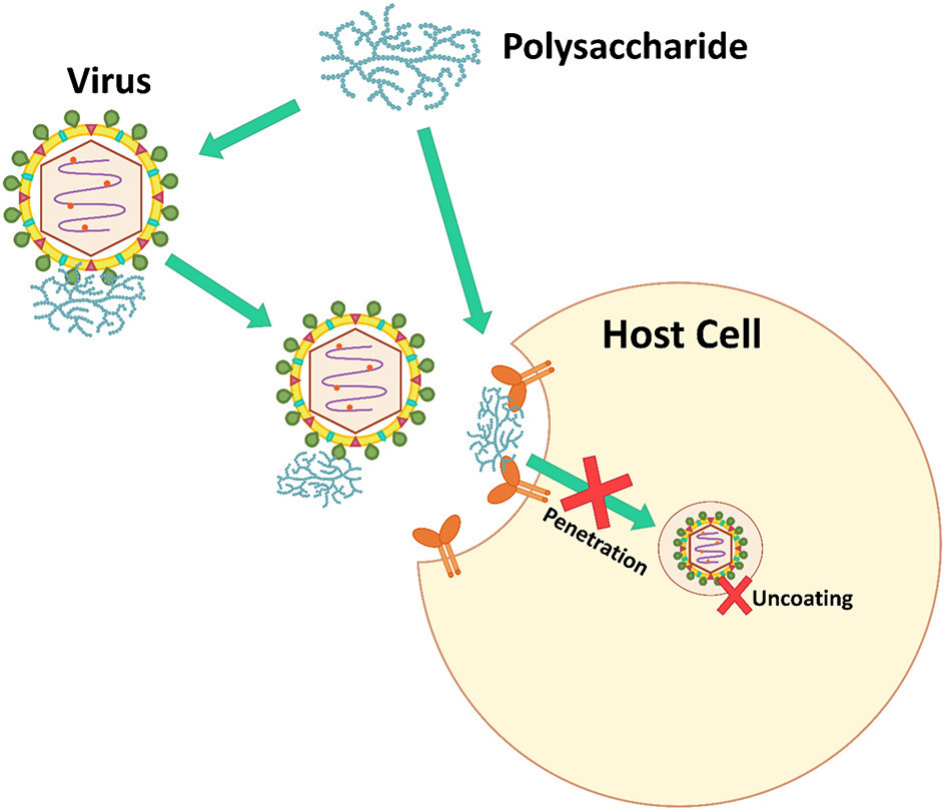
The inhibition mechanism of inhibiting virus adsorption and penetration.

Viruses bind to a host cell surface using electrostatic interactions. Some microbial polysaccharides mimic virus particles. Microbial polysaccharides, especially sulfated polysaccharides, are strongly anionic and bind to the positively charged host cell receptors blocking virus attachment, which prevents virus infection (Figure 3) (7). Additionally, some microbial polysaccharides are able to prevent the allosteric process of viral protein formation and/or virus internalization and uncoating steps (106).

Many microbial polysaccharides act at this step. For example, a polysaccharide SP-2a from *S. patens* showed ≥80% inhibition against all strains of HSV-1 when added during virus adsorption (71). Fucoidans from a brown alga, *Padina tetrastromatica* showed the highest percentage (>70%) inhibition against HSV-1 during the virus adsorption period (37). Human influenza virus H1-364 was blocked by sulfated polysaccharides from *Gracilaria lemaneiformis*, a red alga, at virus adsorption and replication on host cells. The sulfated polysaccharides inhibited against the virus at ≥60% during virus adsorption and replication, while these polysaccharides were not effective at the virus release step. The polysaccharides at 62.5 μg·mL^−1^ showed 83.5 and 83.0% inhibition against human influenza virus H1-364 at the virus adsorption and replication steps, respectively (59).

### Inhibiting Viral Genome Replication and Protein Synthesis

**Figure 4:**
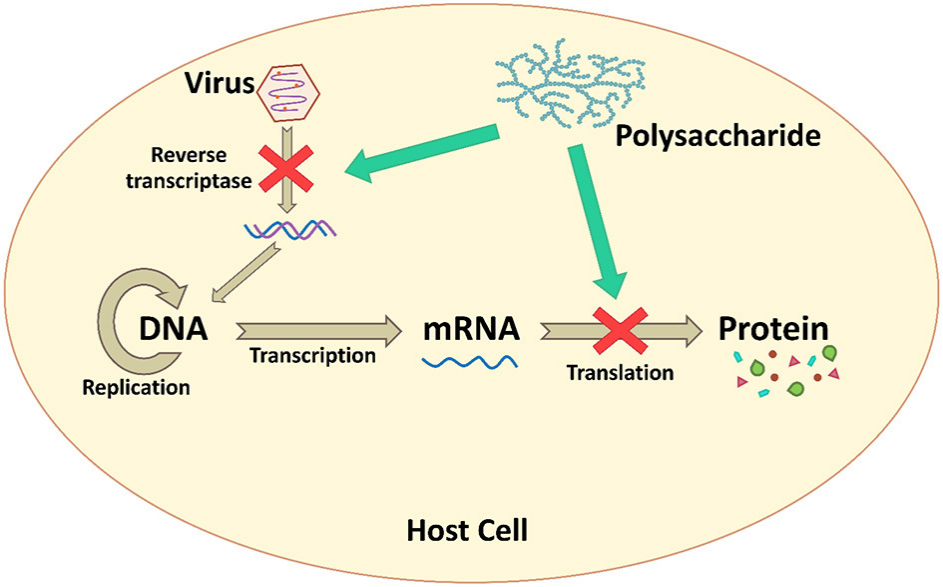
The inhibition mechanism by interfering with viral genome replication and protein synthesis.

Microbial polysaccharides, especially the low MW polysaccharides, show antiviral effects on infected host cells. They interfere directly with enzymes associated with the viral replication and inhibit other intracellular targets (107) as presented in Figure 4. Carrageenans are sulfated polysaccharide that are available from most of red seaweeds. These polysaccharides show a broad-spectrum antiviral activity. González et al. (87) reported that carrageenan inhibited HSV-1 at viral protein synthesis. When carrageenan was added 1 h after HSV-1 infection, viral proteins were not detected, whereas when carrageenan was added immediately, viral proteins were detected. Furthermore, polysaccharide GFP1 from *G. frondosa*, which was composed of glucose and fucose with a MW of 40.5 kDa, acted on viral replication and protein synthesis against EV71. The GFP1 was effective in inhibiting EV71 when it was added before or shortly after the viral inoculation. The viral RNA synthesis and VP1 protein were suppressed in a dose-dependent manner (82).

### Modulating Host Antiviral Immune Responses

**Figure 5:**
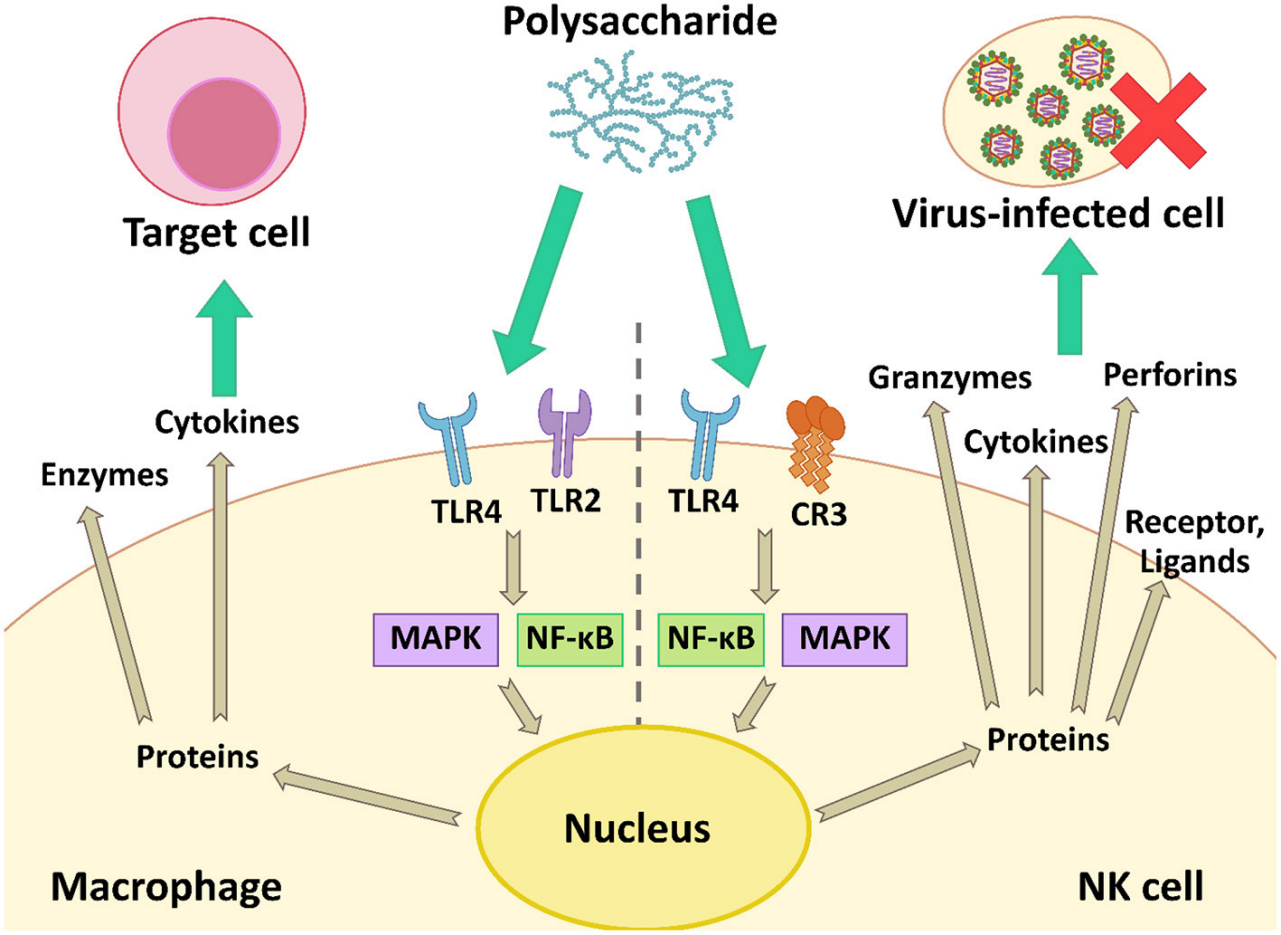
The modulation of the antiviral immune response by activation of macrophage and NK cell using the NF-κB and MAPK signaling pathways.

During virus infection in animals, the body induces the immune responses to defend against viral infection. The responses regulate immune cells such as natural killer (NK) cells and macrophages, and increase the production of cytokines, i.e., the type I interferon system (IFN-α/β system) (36). The microbial polysaccharides interact with cell receptors on the macrophage and NK cell, and then activate the cells using the nuclear factor kappa B (NF-κB) and the mitogen-activated protein kinase (MAPK) signaling pathways. These proteins are inducible factors, which increases the gene expression of various cytokines, chemokines, enzymes, and other proteins involving both innate and adaptive immunity (27). The IFN secreted from activated immune cells triggers activation of other immune cells including NK cells, macrophages, and T-cell lymphocytes, which have important roles in the host immune system and antiviral responses. Meanwhile, microbial polysaccharides can activate NK cells that non-specifically kill virus-infected cells by secreting perforins and granzymes (Figure 5).

Several polysaccharides can enhance the antiviral immune responses, thus reducing the number of virus particles and the severity of diseases. For example, an EPS extracted from *S. thermophilus* ST538 was able to induce the expression of IFN-β, IL-6, and CXCL10 in response to TLR3 stimulation. These immune factors are associated with antiviral immune responses, which induce the recruitment and activation of immune cells to struggle pathogens (98). Moreover, *L. delbrueckii* OLL1073R-1 produced immunomodulatory EPS. These EPS activated TLR3 and induced the expression of IFN-α, IFN-β, MxA, and RNase L. The latter two factors are known as antiviral factors (77). Polysaccharides also showed immune-enhancing activity. Ren et al. (83) reported that LNT-I from *L. edodes* mycelia could modulate the immune response by up-regulating the expression of IFN-1 and IFN-γ to prevent IHNV infection. In addition, an acidic polysaccharide (APS) from *Cordyceps militaris* enhanced TNF-α, IFN-γ, and nitric oxide (NO) production, and induced the expression of several cytokines: IL-1β, IL-6, IL-10, and TNF-α. These cytokines have the potential to prevent influenza A virus infection (39). Cao et al. (64) also reported a polysaccharide from *L. japonica* which could increase IFN-α secretion in a dose-dependent manner. The IFN-α level was 144 pg·mL^−1^ when cells were treated with 1000 μg·mL^−1^ of polysaccharide.

## Factors Influencing the Antiviral Activity

Polysaccharides derived from different sources showed several unique characteristics, properties, and bioactivities at different levels. Table 2 shows various microbial polysaccharides and/or polysaccharide fractions with different characteristics. Their MW, compositions, functional groups, and structural conformations including type of linkage and degree of branching associate with their biological properties, especially antiviral and immunomodulatory activities. Moreover, extraction and purification methods affect the compositions of polysaccharides; therefore, these factors also influence biological activities of the polysaccharides (110).

**Table 2:** Some microbial polysaccharides from various sources with different characteristics and bioactivities.

**Name or fraction**	**Source**	**Average molecular weight (kDa)**	**Polysaccharide Characterisation**	**Observations**	**References**
Acidic polysaccharide (APS)	*Cordyceps militaris*	576	Containing: D-galactose, L-arabinose, D-xylose, L-rhamnose, and D-galacturonic acid; Linkage: Ara*f*-(1 →, → 5)-Ara*f*-(1 →, → 4)-Gal*p*-(1 → and → 4)-GalA*p*-(1 → residues	Influenza A virus titres in the bronchoalveolar lavage fluid and the lung of mice were decreased. The APS increased the production of TNF-α, IFN-γ, and nitric oxide (NO)	(39)
EPS0142	*Lactobacillus plantarum* JLK0142	134	Containing: glucose and galactose in an approximate molar ratio of 2.13:1.06	*In vitro*, EPS0142 improved the phagocytic activity and induced NO secretion*In vivo*, sIgA and cytokines (IL-2 and TNF-α) were increased	(108)
EPS-1	*Monascus purpureus*	69.7	Containing: mannose, glucose and galactose in the molar ratio of 8:1:11; Linkage: a backbone of 5-β-D-Gal*f* and 2-β-D-Man*p* with other sugar residues including 2,6-β-D-Gal*f*, T-α-D-Man*p*, 6-α-D-Man*p*, and 3,5-β-D-Gal*f*	EPS-1 induced cytokines secretion, including IL-6, TNF-α, and IL-10, and up-regulated the related mRNA expression levels.	(109)
FucoidanST-F	*Sargassum trichophyllum*	19.8	Containing: fucose (79.1 mol%) and galactose (19.9 mol%), and its sulfate content was 25.5%; Linkage: terminal, 1,4- and 1,3-linked fucose and terminal, 1,2- and 1,6-linked galactose residues	ST-F showed anti-HSV-2 activity with the IC_50_ of 18 μg·mL^−1^	(72)
Fucoidans SHAP-1 and SHAP-2	*Sargassum henslowianum*	655 (SHAP-1) and 589 (SHAP-2)	Containing: fucose and galactose at a ratio of 3:1; sulfate content of 31.9%; Linkage: a backbone of α-(1 → 3)-linked L-Fuc*p* and a side chain of terminally linked α-L-Fuc*p* and α-D-Gal*p* residues, and (1 → 2)-, (1 → 6)-, and (1 → 2,6)-linked β-D-Gal*p* residues	Both SHAP-1 and SHAP-2 showed antiviral activity against HSV-1 and HSV-2. The IC_50_ values of SHAP-1 and SHAP-2 against HSV-1 were 0.89 and 0.82 μg·mL^−1^, respectively, whereas both as low as 0.48 μg·mL^−1^ against HSV-2.	(84)
GFP1	*Grifola frondosa*	40.5	Containing: glucose and fucose; a backbone of 1,6-β-β-glucan and a single 1,3-α-β-fucopyranosyl side-branching unit	GFP1 could inhibit EV71 replication in Vero cells and suppress viral VP1 protein expression	(82)
Lentinan LNT-1	*Lentinus edodes*	379	Containing: glucose, mannose and galactose with the molar ratio of 19.26:1.20:1.00; Linkage: β-(1 → 3)-glucan backbone with -(1 → 6)-glucosyl side-branching units terminated by mannosyl and galactosyl residue	Lentinan directly inactivate IHNV and modulate immune responses by induce the expression of IFN-1 and IFN-γ, and reduce the expression of TNF-α, IL-2 and IL-11 in EPC cells	(83)
Naviculan	*Navicula directa*	222	Containing: fucose (26.6%), xylose (25.0%), galactose (20.7%), mannose (13.1%), and rhamnose (8.7%)	Naviculan had a broad antiviral spectrum against HSV-1, HSV-2, and Influenza A virus with the IC_50_ values of 14, 7.4, and 170 mg·mL^−1^, respectively.	(67)
Nostoflan	*Nostoc flagelliforme*	211	→ 4)-β-D-Glcp-(1 → 4)-D-Xylp-(1 → and → 4)-[β-D-GlcAp-(1 → 6)-]-β-D-Glcp-(1 → 4)-D-Galp-(1 →	Nostoflan showed broad antiviral activity against HSV-1, HSV-2, HCMV, and Influenza A virus	(79)
Sulfated galactofucan (SJ-D-S-H) and glucuronomannan (Gn)	*Saccharina japonica*	13.7 (SJ-D-S-H) and 7.0 (Gn)	**SJ-D-S-H** Containing: 36% fucose, 10% galactose, and 21% sulfate **Gn** Containing: alternating 1, 4-linked β-D-GlcA*p* residues and 1, 2-linked α-D-Man*p* residues	Both SJ-D-S-H and Gn showed good binding ability to SARS-CoV-2 spike glycoproteins. The IC_50_ values were 27 and 231 nM, respectively	(69)
Sulfated polysaccharide (CIF2)	*Cystoseira indica*	1,150	Containing: mainly fucose (24.4%), glucose (21.3%), mannose (20.6%), galactose (16.7%), rhamnose (11.3%), and xylose (5.8%); Linkage: (1 → 3)-fucopyranose, (1 → 3) and (1 → 6)-galactopyranose residues	*In vitro*, CIF2 increased the release of nitric oxide and inflammatory cytokines including TNF-α, IL-1β, IL-6, and IL-10 from RAW264.7 cells	(54)

### Sulfate Content

Several studies have reported that sulfated polysaccharides could exhibit several biological activities (antiviral, anticancer, antioxidant, and immunomodulatory activities), so the sulfate contents could be an important factor affecting antiviral and other bioactivities. Sulfation has been used for enhancing various biological activities of polysaccharides (111, 112). For example, a marine *Pseudomonas* sp. WAK-1 produces extracellular glycosaminoglycan (A1) and sulfated polysaccharide (A2) with antiviral activity. Matsuda et al. (80) modified the polysaccharides by over-sulfation using a dicyclohexyl-carbodiimide-mediated reaction. The over-sulfated polysaccharides were called A1S and A2S, respectively. These 4 compounds showed antiviral activity against influenza A virus with EC_50_ values of >100, >100, 11.0, and 2.9 μg·mL^−1^, respectively. From the results, over-sulfated polysaccharides (A1S and A2S) showed higher antiviral activity against influenza A virus than the natural polysaccharides (A1 and A2). Moreover, a xylogalactofucan (sulfated polysaccharide) from a brown alga *Sphacelaria indica* also exhibited antiviral activity against HSV-1. The sulfate contents of the polysaccharide affected the antiviral property. The sulfate content of the purified polysaccharide was 4% (w/w). Bandyopadhyay et al. (113) chemically modified the xylogalactofucan produced derivatives with up to 7% (w/w). The IC_50_ values of natural and artificially over-sulfated polysaccharides were 1.3 and 1.5 μg·mL^−1^, respectively, while a desulfated derivative of the polysaccharide had no effect on HSV-1. Furthermore, Ponce et al. (86) also reported that the level of sulfated esters in sulfated galactofucans extracted from a brown alga *Scytosiphon lomentaria* was an important factor influencing the antiviral activity. The whole extract (A) of *S. lomentaria* was fractionated to yield fractions A0, A5, A10, A20, A30, and A40, with different components, MW, and monosaccharide composition. A0 was soluble and the fraction A5 was an uronofucoidan. A10–A40 were galactofucans and showed antiviral activity against HSV-1 and HSV-2 with IC_50_ values in the range 0.76–10.0 μg·mL^−1^. Among the 4 galactofucan fractions, A30 (pure galactofucan) contained the highest sulfate content (29.5% SO_3_Na) and the lowest uronate content (1.8%). A30 showed the strongest antiviral activity against HSV-1 and HSV-2 with IC_50_ values of 0.76 and 1.3 μg·mL^−1^, respectively. Therefore, the low content of uronic acids and the high content of sulfate was associated with the antiviral activity of these polysaccharides. In conclusion, sulfate content is an important factor influencing biological activities. Adding sulfate groups into polysaccharide structures led to enhance bioactivities, whereas desulfation decreased their bioactivities.

### Molecular Weight

The MW of polysaccharides also influenced their biological properties. Polysaccharides with low MW could easily pass through target cells to act inside the cells. Moreover, the low MW polysaccharides might bind better to cell receptors to inactivate or activate the target cells (114). Some polysaccharides with lower MW showed high biological activities, but some polysaccharides with higher MW were better. For example, Surayot et al. (26) reported the effect of MW of an EPS from *Weisella confusa* on immunomodulatory activity. The EPS with low MW (≤70 × 10^3^ Da) could stimulate RAW264.7 cells to induce NO and production of various cytokines such as TNF-α, IL-1β, IL-6, and IL-10, but the native EPS (MW of ~506 × 10^6^ Da) had no immunomodulatory activity. In addition, Ponce et al. (86) reported that the galactofucan fraction A30 had the lowest MW (~8.5 kDa) and the low MW might be another factor influencing the antiherpes activity against HSV-1 and HSV-2. On the contrary, high molecular weight carrageenans from different rea algae (*Chondrus armatus, Kappaphycus alvarezii*, and *Tichocarpus crinitus*) had effective antiviral activity (115). The different carrageenans with molecular weight of 250, 390, and 400 kDa, respectively show antiviral activity with 88, 85, and 77%, respectively. While low molecular weight (LMW) derivatives (1.2–3.5 kDa) were obtained from different depolymerization methods. The LMW derivatives showed low antiviral properties (28–54%) compared to the native polysaccharides. Therefore, the antiviral activity of these polysaccharides depended on their molecular weight (115).

### Enhancement of Bioactivities

To enhance the biological activities, natural microbial polysaccharides need molecular modification of their structure, size, and functional groups to optimize activity (114). The MW can be reduced using external energy and/or specific enzymes to break glucoside chains. Using ultrasonic disruption and microwave exposure to reduce the MW are “physical modification,” whereas the enzymatic degradation is “biological modification” (114). For instance, Surayot et al. (26) hydrolyzed an EPS from *W. confusa* TISTR 1498 using HCl and heating in hot water or in a microwave oven. The low MW products could induce production of cytokines from RAW264.7 macrophage cells. Bioactivities were also enhanced by changing the functional substituents of polysaccharides, which is “chemical modification,” such as alkylation, sulfation, sulfonation, phosphorylation, carboxymethylation, and selenization (114).

## The Potential Uses of Microbial Polysaccharides to Prevent Viral Diseases

Microbial polysaccharides showed various bioactivities, while almost always having any significant side-effects, yet are biodegradable, biocompatible, and cost-effective. Microbial and algal polysaccharides may be applied as drug resistance solutions. These polysaccharides can combine with other antiviral drugs for preventing drug-resistance strains (110). In addition to the prevention of viral infections, these polysaccharides also prevent recurrence of latent viruses. For example, calcium spirulan (Ca-SP) derived from *Spirulina platensis* was developed as microalgal cream, which effectively prevented the recurrence of HSV-1 (116). Therefore, the bioactive polysaccharides may be used to prevent viral diseases and reduce the risks of diseases, especially COVID-19.

SARS-CoV-2 has an S-protein on its envelope and the protein has an important role with binding to a host cell receptor (ACE2) (8). Heparin, heparan sulfates, and other sulfated polysaccharides can bind tightly to the S-protein *in vitro* (117). The binding inhibits viral infection. Other microbial polysaccharides showed immunomodulatory properties that stimulated the immune system to prevent SARS-CoV-2 infection. Several microbes can produce sulfated polysaccharides. Beneficial sulfated polysaccharides might be produced from natural microbial polysaccharides (114). Type I IFN, including IFN-α, -β, -ε, -κ, -ω, -δ, -ζ, and -τ, are essential cytokines for antiviral immune responses. Type I IFN can induce antiviral responses within infected and neighboring cells that block the spread of virus particles. They activate both innate and adaptive immune responses that promote NK cell functions and antibody production (118, 119). Hadjadj et al. (120) reported that most severe COVID-19 patients had a low type I IFN response (No IFN-β and low IFN-α production and activity). Many microbial and algal polysaccharides induced type I IFN production *in vivo*. Thus, these polysaccharides might be applied to modulate the immune system in both patients and healthy people. In severe COVID-19 cases, aggressive inflammatory responses were found and the inflammation caused tissue damage in many organs (121). Some microbial polysaccharides showed anti-inflammatory activity. These polysaccharides inhibited the production of pro-inflammatory cytokines including TNF-α, IL-1β, IL-6, and IL-8 (122–124).

ACE2 receptors are expressed by several tissues and organs as described above, especially the respiratory and gastrointestinal tracts. Microbial polysaccharides with antiviral activity can be used as a nasal spray, metered dose inhaler, or delivered orally to prevent the binding of SARS-CoV-2 (117). Several natural polysaccharides have been designed as nanomaterials for drug delivery systems, such as antiviral agent. These nanomaterials may be not only used to treat the virus, but also to modulate the immune responses (7).

Algal, bacterial, and fungal polysaccharides and sulfated polysaccharides showed pharmaceutical properties due to their biological activities as mentioned above. These polysaccharides could be used as bioactive supplements in foods and could enrich nutritional quality. Indeed, some of these polysaccharides have been granted as GRAS status by the US FDA, so they can consume to enhance immune response and reduce the severity of viral diseases, especially COVID-19 (117). Some microbial polysaccharides have prebiotic properties, which enhance the proliferation of beneficial intestinal microflora, especially *Bifidobacterium* spp. (125). In addition, some algal polysaccharides (alginate and laminaran) could be fermented by gut microbiota and promoted the growth of *Bacteroides, Bifidobacterium*, and *Lactobacillus* species (126). When microbial polysaccharides were consumed, they could enhance the host's immune response and modulate the microbial community (microflora). The microbes degrade the polysaccharides into short-chain fatty acids (SCFA) such as acetic, propionic, and butyric acids. SCFA show benefit for the maintenance intestinal cells and modulating of the immune system (27). Microbial polysaccharides have the potential to be bioactive ingredients that can be added into foods or food products to enhance the nutritional quality of foods by modulating consumers' immune response. Therefore, the biological activities of foods supplemented with these polysaccharides should be investigated. The intake of foods with bioactive polysaccharides in patients and healthy people to prevent viruses and/or reduce the adverse symptoms needs further study.

## Conclusion

Microorganisms produce various types of polysaccharides with unique characteristics and can be produced on a large scale with controllable conditions. Several microbial/algal polysaccharides show bioactivities, especially antiviral and immunomodulatory activities. They have strong antiviral effects by interfering with the life cycle of viruses and/or modulating host immune responses, which may benefit patients infected with COVID-19. Polysaccharides and sulfated polysaccharides from different microorganism and algae species have different characteristics and levels of bioactivities. Their constituents, structural conformations, MW, and functional groups significantly influence their bioactivities. To enhance their activities, physical, chemical, or biological modifications might be beneficial. The microbial polysaccharides have potential uses as adjuvants for antiviral vaccines and micro- and/or nano-particles for drug delivery systems. Some sulfated polysaccharides obtained from microbes and algae have been approved as GRAS, which may be used as bioactive ingredients adding in food products to prevent viruses. Many microbial polysaccharides are safe, biocompatible, biodegradable, and easily available. Therefore, the intake of proper dosage of the right polysaccharides may modulate physiological functions to prevent viral diseases and decrease their damage. They may be an alternative therapy to treat COVID-19 patients. In the future, the development of polysaccharides as functional food products should be explored. For foods supplemented with bioactive polysaccharides, more pharmaceutical investigations and clinical evidence are required to analyze their antiviral and immune-enhancing effects. The mechanisms that occur in the food products against viral infections should also be further investigated.

## Author Contributions

PS, TC, CT, and SY contributed to conception and supervised the project. WC, NL, KJ, and SS contributed in doing literature searches and wrote the manuscript draft. YP, PR, SW, JR, FB, and PS equally revised and approved the manuscript. All authors have read and approved the final draft manuscript.

## Funding

The authors gratefully acknowledge the financial support from the National Research Council of Thailand (NRCT) through the Royal Golden Jubilee Ph.D. Program, Thailand (grant no. PHD/0185/2560) to WC and PS. Additionally, this work was also partially financially supported along with in-kind support by the Biotechnology Program of the Graduate School of Chiang Mai University; the Cluster of Agro Bio-Circular-Green Industry (Agro BCG) Faculty of Agro-Industry; and Chiang Mai University.

## Conflict of Interest

The authors declare that the research was conducted in the absence of any commercial or financial relationships that could be construed as a potential conflict of interest.

## Publisher's Note

All claims expressed in this article are solely those of the authors and do not necessarily represent those of their affiliated organizations, or those of the publisher, the editors and the reviewers. Any product that may be evaluated in this article, or claim that may be made by its manufacturer, is not guaranteed or endorsed by the publisher.

## Abbreviations

ACE2: Angiotensin-converting enzyme 2
ACV: Acyclovir
APS: Acidic polysaccharide
BoHV-1: Bovine herpesvirus type 1
BoHV-1: Bovine herpes virus type 1
CoV: Coronaviruses
COVID-19: Coronavirus disease 2019
Cox-B3: Coxsackie B virus type 3
CVB3: Coxsackie virus B3
CXCL10: C-X-C motif chemokine 10
ED_50_: 50% effective dose
EMCV: Encephalomyocarditis virus
EPS: Exopolysaccharides
EV71: Enterovirus 71
FCoV: Feline coronavirus
FCV: Feline calicivirus
FDA: US Food and Drug Administration
FHV-1: Feline herpesvirus 1
FIV: Feline influenza
FPV: Feline panleukopenia
HAdV-5: Human adenovirus type 5
HAV: Hepatitis A virus
HCMV: Human cytomegalovirus
HHV-6: Human herpesvirus type 6
HIV: Human immunodeficiency virus
HSV: Herpes simplex virus
IC_50_: 50% inhibitory concentration
ICP: Intracellular polysaccharides
ICTV: International Committee on Taxonomy of Viruses
IFN: Interferon
IHNV: Hematopoietic necrosis virus
IL: Interleukin
KHV: Koi herpesviruses
LAB: Lactic acid bacteria
MAPK: Mitogen-activated protein kinase
MERS-CoV: Middle East respiratory syndrome
MW: Molecular weight
NDV: Newcastle disease virus
NF-κB: Nuclear factor kappa B
NK: Natural killer cells
NO: Nitric oxide
PBMC: Peripheral blood mononuclear cells
PV-1: Poliovirus type 1
SARS-CoV-2: Severe acute respiratory syndrome coronavirus 2
SCFA: Short-chain fatty acids
SuHV-1: Suid herpesvirus type 1
TLR3: Toll-like receptor 3
TMPRSS2: Transmembrane protease serine protease 2
TMV: Tobacco mosaic virus
VSV: Vesicular stomatitis virus
VZV: Varicella zoster virus
WHO: World Health Organization.

## References

[1] Dimmock NJ Easton AJ Leppard KN. Introduction to Modern Virology. Chichester: John Wiley & Sons (2016). 544 p.

[2] World Health Organization. Rolling Updates on Coronavirus Disease (COVID-19) (2021). Available online at: https://www.who.int/emergencies/diseases/novel-coronavirus-2019/events-as-they-happen (accessed May 3, 2021).

[3] World Health Organization. WHO Coronavirus (COVID-19) Dashboard (2021). Available online at: https://covid19.who.int/ (accessed August 9, 2021).

[4] Mohan SV Hemalatha M Kopperi H Ranjith I Kumar AK. SARS-CoV-2 in environmental perspective: occurrence, persistence, surveillance, inactivation and challenges. Chem Eng J. (2021) 405:126893. 10.1016/j.cej.2020.126893 32901196 PMC7471803

[5] Chen B Tian EK He B Tian L Han R Wang S. Overview of lethal human coronaviruses. Signal Transduct Target Ther. (2020) 5:89. 10.1038/s41392-020-0190-2 32533062 PMC7289715

[6] V'Kovski P Kratzel A Steiner S Stalder H Thiel V. Coronavirus biology and replication: implications for SARS-CoV-2. Nat Rev Microbiol. (2020) 19:155–70. 10.1038/s41579-020-00468-6 33116300 PMC7592455

[7] Chen X Han W Wang G Zhao X. Application prospect of polysaccharides in the development of anti-novel coronavirus drugs and vaccines. Int J Biol Macromol. (2020) 164:331–43. 10.1016/j.ijbiomac.2020.07.106 32679328 PMC7358770

[8] Cevik M Kuppalli K Kindrachuk J Peiris M. Virology, transmission, and pathogenesis of SARS-CoV-2. BMJ. (2020) 371:m3862. 10.1136/bmj.m3862 33097561

[9] Di Nardo M van Leeuwen G Loreti A Barbieri MA Guner Y Locatelli F. A literature review of 2019 novel coronavirus (SARS-CoV2) infection in neonates and children. Pediatr Res. (2020) 89:1101–8. 10.1038/s41390-020-1065-5 32679582

[10] Jia HP Look DC Shi L Hickey M Pewe L Netland J. ACE2 receptor expression and severe acute respiratory syndrome coronavirus infection depend on differentiation of human airway epithelia. J Virol. (2005) 79:14614–21. 10.1128/JVI.79.23.14614-14621.2005 16282461 PMC1287568

[11] Li MY Li L Zhang Y Wang XS. Expression of the SARS-CoV-2 cell receptor gene ACE2 in a wide variety of human tissues. Infect Dis Poverty. (2020) 9:45. 10.1186/s40249-020-00662-x 32345362 PMC7186534

[12] Xu H Zhong L Deng J Peng J Dan H Zeng X. High expression of ACE2 receptor of 2019-nCoV on the epithelial cells of oral mucosa. Int J Oral Sci. (2020) 12:8. 10.1038/s41368-020-0074-x 32094336 PMC7039956

[13] Kelleni MT. Tocilizumab, remdesivir, favipiravir, and dexamethasone repurposed for COVID-19: a comprehensive clinical and pharmacovigilant reassessment. SN Compr Clin Med. (2021) 3:919–23. 10.1007/s42399-021-00824-4 33644693 PMC7894610

[14] Beigel JH Tomashek KM Dodd LE Mehta AK Zingman BS Kalil AC. Remdesivir for the treatment of covid-19 - final report. N Engl J Med. (2020) 383:1813–26. 10.1056/NEJMoa2007764 32445440 PMC7262788

[15] Centers for Disease Control and Prevention. Treatments Your Healthcare Provider Might Recommend if You Are Sick (2020). Available online at: https://www.cdc.gov/coronavirus/2019-ncov/your-health/treatments-for-severe-illness.html (accessed December 24, 2020).

[16] WHO Solidarity Trial Consortium. Repurposed antiviral drugs for COVID-19—interim WHO solidarity trial results. N Engl J Med (2021) 384:497–511. 10.1056/NEJMoa2023184 33264556 PMC7727327

[17] Centers for Disease Control and Prevention. Different COVID-19 Vaccines (2020). Available online at: https://www.cdc.gov/coronavirus/2019-ncov/vaccines/different-vaccines.html (accessed December 24, 2020).

[18] Thirumdas R Kothakota A Pandiselvam R Bahrami A Barba FJ. Role of food nutrients and supplementation in fighting against viral infections and boosting immunity: a review. Trends Food Sci Technol. (2021) 110:66–77. 10.1016/j.tifs.2021.01.069 33558789 PMC7857987

[19] Abdelkebir R Alcántara C Falcó I Sánchez G Garcia-Perez JV Neffati M. Effect of ultrasound technology combined with binary mixtures of ethanol and water on antibacterial and antiviral activities of *Erodium glaucophyllum* extracts. Innov Food Sci Emerg Technol. (2019) 52:189–96. 10.1016/j.ifset.2018.12.009

[20] Galanakis CM Aldawoud TMS Rizou M Rowan NJ Ibrahim SA. Food ingredients and active compounds against the coronavirus disease (COVID-19) pandemic: a comprehensive review. Foods. (2020) 9:1701. 10.3390/foods9111701 33233560 PMC7699782

[21] Galanakis CM Rizou M Aldawoud TMS Ucak I Rowan NJ. Innovations and technology disruptions in the food sector within the COVID-19 pandemic and post-lockdown era. Trends Food Sci Technol. (2021) 110:193–200. 10.1016/j.tifs.2021.02.002 PMC9759022 36567851

[22] El Khadem HS. Carbohydrates. In: Meyers RA, editor. Encyclopedia of Physical Science and Technology, 3rd Edn. Amsterdam, Netherlands: Elsevier B.V (2003). p. 369–416.

[23] Barbosa JR de Carvalho RN Junior. Polysaccharides obtained from natural edible sources and their role in modulating the immune system: biologically active potential that can be exploited against COVID-19. Trends Food Sci Technol. (2021) 108:223–35. 10.1016/j.tifs.2020.12.026 33424125 PMC7781518

[24] Shi Y Xiong Q Wang X Li X Yu C Wu J. Characterization of a novel purified polysaccharide from the flesh of *Cipangopaludina chinensis*. Carbohydr Polym. (2016) 136:875–83. 10.1016/j.carbpol.2015.09.062 26572424

[25] Surin S Surayot U Seesuriyachan P You SG Phimolsiripol Y. Antioxidant and immunomodulatory activities of sulphated polysaccharides from purple glutinous rice bran (*Oryza sativa* L.). Int J Food Sci. (2018) 53:994–1004. 10.1111/ijfs.13674

[26] Surayot U Wang J Seesuriyachan P Kuntiya A Tabarsa M Lee Y. Exopolysaccharides from lactic acid bacteria: structural analysis, molecular weight effect on immunomodulation. Int J Biol Macromol. (2014) 68:233–40. 10.1016/j.ijbiomac.2014.05.005 24820155

[27] Chaisuwan W Jantanasakulwong K Wangtueai S Phimolsiripol Y Chaiyaso T Techapun C. Microbial exopolysaccharides for immune enhancement: fermentation, modifications and bioactivities. Food Biosci. (2020) 35:100564. 10.1016/j.fbio.2020.100564

[28] Nwodo UU Green E Okoh AI. Bacterial exopolysaccharides: functionality and prospects. Int J Mol Sci. (2012) 13:14002–15. 10.3390/ijms131114002 23203046 PMC3509562

[29] Wang J Salem DR Sani RK. Extremophilic exopolysaccharides: a review and new perspectives on engineering strategies and applications. Carbohydr Polym. (2019) 205:8–26. 10.1016/j.carbpol.2018.10.011 30446151

[30] Rana S Upadhyay LSB. Microbial exopolysaccharides: synthesis pathways, types and their commercial applications. Int J Biol Macromol. (2020) 157:577–83. 10.1016/j.ijbiomac.2020.04.084 32304790

[31] Andrew M Jayaraman G. Marine sulfated polysaccharides as potential antiviral drug candidates to treat Corona Virus disease (COVID-19). Carbohydr Res. (2021) 505:108326. 10.1016/j.carres.2021.108326 34015720 PMC8091805

[32] Muthukumar J Chidambaram R Sukumaran S. Sulfated polysaccharides and its commercial applications in food industries—A review. J Food Sci Technol. (2021) 58:2453–66. 10.1007/s13197-020-04837-0 34194082 PMC8196116

[33] Ipper NS Cho S Lee SH Cho JM Hur JH Lim CK. Antiviral activity of the exopolysaccharide produced by *Serratia* sp. strain Gsm01 against Cucumber mosaic virus. J Microbiol Biotechnol. (2008) 18:67–73. 18239419

[34] Aoki M Tan M Fukushima A Hieda T Kubo S Takabayashi M. Antiviral substances with systemic effects produced by Basidiomycetes such as *Fomes fomentarius*. Biosci Biotechnol Biochem. (1993) 57:278–82. 10.1271/bbb.57.278 27314782

[35] Tian J Hu X Liu D Wu H Qu L. Identification of *Inonotus obliquus* polysaccharide with broad-spectrum antiviral activity against multi-feline viruses. Int J Biol Macromol. (2017) 95:160–7. 10.1016/j.ijbiomac.2016.11.054 27865960 PMC7185483

[36] Chen L Huang G. The antiviral activity of polysaccharides and their derivatives. Int J Biol Macromol. (2018) 115:77–82. 10.1016/j.ijbiomac.2018.04.056 29654857

[37] Karmakar P Pujol CA Damonte EB Ghosh T Ray B. Polysaccharides from *Padina tetrastromatica*: structural features, chemical modification and antiviral activity. Carbohydr Polym. (2010) 80:513–20. 10.1016/j.carbpol.2009.12.014

[38] Huleihel M Ishanu V Tal J Arad SM. Antiviral effect of red microalgal polysaccharides on *Herpes simplex* and *Varicella zoster* viruses. J Appl Phycol. (2001) 13:127–34. 10.1023/A:1011178225912

[39] Ohta Y Lee JB Hayashi K Fujita A Park DK Hayashi T. *In vivo* anti-influenza virus activity of an immunomodulatory acidic polysaccharide isolated from *Cordyceps militaris* grown on germinated soybeans. J Agric Food Chem. (2007) 55:10194–9. 10.1021/jf0721287 17988090

[40] Komatsu T Kido N Sugiyama T Yokochi T. Antiviral activity of acidic polysaccharides from *Coccomyxa gloeobotrydiformi*, a green alga, against an *in vitro* human influenza A virus infection. Immunopharmacol Immunotoxicol. (2013) 35:1–7. 10.3109/08923973.2012.710636 22856509

[41] Biliavska L Pankivska Y Povnitsa O Zagorodnya S. Antiviral activity of exopolysaccharides produced by lactic acid bacteria of the genera *Pediococcus, Leuconostoc* and *Lactobacillus* against human adenovirus type 5. Medicina. (2019) 55:519. 10.3390/medicina55090519 31443536 PMC6780409

[42] Nguyen TL Chen J Hu Y Wang D Fan Y Wang J. *In vitro* antiviral activity of sulfated *Auricularia auricula* polysaccharides. Carbohydr Polym. (2012) 90:1254–8. 10.1016/j.carbpol.2012.06.060 22939338

[43] Abu-Galiyun E Huleihel M Levy-Ontman O. Antiviral bioactivity of renewable polysaccharides against *Varicella Zoster*. Cell Cycle. (2019) 18:3540–9. 10.1080/15384101.2019.1691363 31724465 PMC6927708

[44] Rechter S König T Auerochs S Thulke S Walter H Dörnenburg H. Antiviral activity of *Arthrospira*-derived spirulan-like substances. Antivir Res. (2006) 72:197–206. 10.1016/j.antiviral.2006.06.004 16884788

[45] Stepanenko LS Maksimov OB Fedoreev SA Miller GG. Polysaccharides of lichens and their sulfated derivatives: antiviral activity. Chem Nat Compd. (1998) 34:337–8. 10.1007/BF02282419

[46] Angelin J Kavitha M. Exopolysaccharides from probiotic bacteria and their health potential. Int J Biol Macromol. (2020) 162:853–65. 10.1016/j.ijbiomac.2020.06.190 32585269 PMC7308007

[47] Delbarre-Ladrat C SC Lebellenger L Zykwinska A Colliec-Jouault S. Exopolysaccharides produced by marine bacteria and their applications as glycosaminoglycan-like molecules. Front Chem. (2014) 2:85. 10.3389/fchem.2014.00085 25340049 PMC4189415

[48] Rosales-Mendoza S García-Silva I González-Ortega O Sandoval-Vargas JM Malla A Vimolmangkang S. The potential of algal biotechnology to produce antiviral compounds and biopharmaceuticals. Molecules. (2020) 25:4049. 10.3390/molecules25184049 32899754 PMC7571207

[49] Ahmadi A Moghadamtousi SZ Abubakar S Zandi K. Antiviral potential of algae polysaccharides isolated from marine sources: a review. Biomed Res Int. (2015) 2015:825203. 10.1155/2015/825203 26484353 PMC4592888

[50] Alboofetileh M Rezaei M Tabarsa M You S. Bioactivities of *Nizamuddinia zanardinii* sulfated polysaccharides extracted by enzyme, ultrasound and enzyme-ultrasound methods. J Food Sci Technol. (2019) 56:1212–20. 10.1007/s13197-019-03584-1 30956301 PMC6423231

[51] Gong G Dang T Deng Y Han J Zou Z Jing S. Physicochemical properties and biological activities of polysaccharides from *Lycium barbarum* prepared by fractional precipitation. Int J Biol Macromol. (2018) 109:611–8. 10.1016/j.ijbiomac.2017.12.017 29222018

[52] Vishwakarma J Vavilala SL. Evaluating the antibacterial and antibiofilm potential of sulphated polysaccharides extracted from green algae *Chlamydomonas reinhardtii*. J Appl Microbiol. (2019) 127:1004–17. 10.1111/jam.14364 31260145

[53] Xu Y Xu J Ge K Tian Q Zhao P Guo Y. Anti-inflammatory effect of low molecular weight fucoidan from *Saccharina japonica* on atherosclerosis in apoE-knockout mice. Int J Biol Macromol. (2018) 118:365–74. 10.1016/j.ijbiomac.2018.06.054 29906534

[54] Bahramzadeh S Tabarsa M You S Li C Bita S. Purification, structural analysis and mechanism of murine macrophage cell activation by sulfated polysaccharides from *Cystoseira indica*. Carbohydr Polym. (2019) 205:261–70. 10.1016/j.carbpol.2018.10.022 30446103

[55] Sanjeewa KKA Kang N Ahn G Jee Y Kim YT Jeon YJ. Bioactive potentials of sulfated polysaccharides isolated from brown seaweed *Sargassum* spp in related to human health applications: a review. Food Hydrocoll. (2018) 81:200–8. 10.1016/j.foodhyd.2018.02.040

[56] Yim JH Kim SJ Ahn SH Lee CK Rhie KT Lee HK. Antiviral effects of sulfated exopolysaccharide from the marine microalga *Gyrodinium impudicum* strain KG03. Mar Biotechnol. (2004) 6:17–25. 10.1007/s10126-003-0002-z 14508657

[57] Cho M Yang C Kim SM You S. Molecular characterization and biological activities of watersoluble sulfated polysaccharides from *Enteromorpha prolifera*. Food Sci Biotechnol. (2010) 19:525–33. 10.1007/s10068-010-0073-3

[58] Cao RA Lee Y You S. Water soluble sulfated-fucans with immune-enhancing properties from *Ecklonia cava*. Int J Biol Macromol. (2014) 67:303–11. 10.1016/j.ijbiomac.2014.03.019 24661888

[59] Chen MZ Xie HG Yang LW Liao ZH Yu J. *In vitro* anti-influenza virus activities of sulfated polysaccharide fractions from *Gracilaria lemaneiformis*. Virol Sin. (2010) 25:341–51. 10.1007/s12250-010-3137-x 20960180 PMC8227872

[60] Kim M Yim JH Kim SY Kim HS Lee WG Kim SJ. *In vitro* inhibition of influenza a virus infection by marine microalga-derived sulfated polysaccharide p-KG03. Antivir Res. (2012) 93:253–9. 10.1016/j.antiviral.2011.12.006 22197247

[61] Santoyo S Plaza M Jaime L Ibañez E Reglero G Señorans J. Pressurized liquids as an alternative green process to extract antiviral agents from the edible seaweed *Himanthalia elongata*. J Appl Phycol. (2010) 23:909–17. 10.1007/s10811-010-9611-x

[62] Wang H Ooi VEC Ang PO. Anti-herpesviral property and mode of action of a polysaccharide from brown seaweed (*Hydroclathrus clathratus*). World J Microbiol Biotechnol. (2010) 26:1703–13. 10.1007/s11274-010-0348-0

[63] Wang H Ooi EV Ang PO Jr. Antiviral polysaccharides isolated from Hong Kong brown seaweed *Hydroclathrus clathratus*. Sci China C Life Sci. (2007) 50:611–8. 10.1007/s11427-007-0086-1 17879058

[64] Cao YG Hao Y Li ZH Liu ST Wang LX. Antiviral activity of polysaccharide extract from *Laminaria japonica* against respiratory syncytial virus. Biomed Pharmacother. (2016) 84:1705–10. 10.1016/j.biopha.2016.10.082 27847204

[65] Yue Y Li Z Li P Song N Li B Lin W. Antiviral activity of a polysaccharide from *Laminaria japonica* against enterovirus 71. Biomed Pharmacother. (2017) 96:256–62. 10.1016/j.biopha.2017.09.117 28987950

[66] Sun T Zhang X Miao Y Zhou Y Shi J Yan M. Studies on antiviral and immuno-regulation activity of low molecular weight fucoidan from *Laminaria japonica*. J Ocean Univ China. (2018) 17:705–11. 10.1007/s11802-018-3794-1

[67] Lee JB Hayashi K Hirata M Kuroda E Suzuki E Kubo Y. Antiviral sulfated polysaccharide from *Navicula directa*, a diatom collected from deep-sea water in Toyama Bay. Biol Pharm Bull. (2006) 29:2135–9. 10.1248/bpb.29.2135 17015966

[68] Talyshinsky MM Souprun YY Huleihel MM. Anti-viral activity of red microalgal polysaccharides against retroviruses. Cancer Cell Int. (2002) 2:8. 10.1186/1475-2867-2-8 12204093 PMC140136

[69] Jin W Zhang W Mitra D McCandless MG Sharma P Tandon R. The structure-activity relationship of the interactions of SARS-CoV-2 spike glycoproteins with glucuronomannan and sulfated galactofucan from *Saccharina japonica*. Int J Biol Macromol. (2020) 163:1649–58. 10.1016/j.ijbiomac.2020.09.184 32979436 PMC7513770

[70] Sun Y Chen X Zhang L Liu H Liu S Yu H. The antiviral property of *Sargassum fusiforme* polysaccharide for avian leukosis virus subgroup J *in vitro* and *in vivo*. Int J Biol Macromol. (2019) 138:70–8. 10.1016/j.ijbiomac.2019.07.073 31306705

[71] Zhu W Chiu LC Ooi VE Chan PK Ang PO Jr. Antiviral property and mechanisms of a sulphated polysaccharide from the brown alga *Sargassum patens* against Herpes simplex virus type 1. Phytomedicine. (2006) 13:695–701. 10.1016/j.phymed.2005.11.003 16427262

[72] Lee J-B Takeshita A Hayashi K Hayashi T. Structures and antiviral activities of polysaccharides from *Sargassum trichophyllum*. Carbohydr Polym. (2011) 86:995–9. 10.1016/j.carbpol.2011.05.059

[73] Chiu YH Chan YL Li TL Wu CJ. Inhibition of Japanese encephalitis virus infection by the sulfated polysaccharide extracts from *Ulva lactuca*. Mar Biotechnol. (2012) 14:468–78. 10.1007/s10126-011-9428-x 22193590

[74] Hayashi T Hayashi K. Calcium spirulan, an inhibitor of enveloped virus replication, from a blue-green alga *Spirulina platensis*. J Nat Prod. (1996) 59:83–7. 10.1021/np960017o 8984158

[75] Reichert M Bergmann SM Hwang J Buchholz R Lindenberger C. Antiviral activity of exopolysaccharides from *Arthrospira platensis* against koi herpesvirus. J Fish Dis. (2017) 40:1441–50. 10.1111/jfd.12618 28422294

[76] Gugliandolo C Spano A Lentini V Arena A Maugeri TL. Antiviral and immunomodulatory effects of a novel bacterial exopolysaccharide of shallow marine vent origin. J Appl Microbiol. (2014) 116:1028–34. 10.1111/jam.12422 24354946

[77] Kanmani P Albarracin L Kobayashi H Iida H Komatsu R Kober AKMH. Exopolysaccharides from *Lactobacillus delbrueckii* OLL1073R-1 modulate innate antiviral immune response in porcine intestinal epithelial cells. Mol Immunol. (2018) 98:253–65. 10.1016/j.molimm.2017.07.009 28800975

[78] Yang Y Song H Wang L Dong W Yang Z Yuan P. Antiviral effects of a probiotic metabolic products against transmissible gastroenteritis Coronavirus. J Probiotics Health. (2017) 5:3. 10.4172/2329-8901.1000184

[79] Kanekiyo K Lee JB Hayashi K Takenaka H Hayakawa Y Endo S. Isolation of an antiviral polysaccharide, nostoflan, from a terrestrial cyanobacterium, *Nostoc flagelliforme*. J Nat Prod. (2005) 68:1037–41. 10.1021/np050056c 16038544

[80] Matsuda M Shigeta S Okutani K. Antiviral activities of marine *Pseudomonas* polysaccharides and their oversulfated derivatives. Mar Biotechnol. (1999) 1:68–73. 10.1007/PL00011753 10373612

[81] Lee SM Kim SM Lee YH Kim WJ Park JK Park YI. Macromolecules isolated from *Phellinus pini* fruiting body: chemical characterization and antiviral activity. Macromol Res. (2010) 18:602–9. 10.1007/s13233-010-0615-9

[82] Zhao C Gao L Wang C Liu B Jin Y Xing Z. Structural characterization and antiviral activity of a novel heteropolysaccharide isolated from *Grifola frondosa* against enterovirus 71. Carbohydr Polym. (2016) 144:382–9. 10.1016/j.carbpol.2015.12.005 27083830

[83] Ren G Xu L Lu T Yin J. Structural characterization and antiviral activity of lentinan from *Lentinus edodes* mycelia against infectious hematopoietic necrosis virus. Int J Biol Macromol. (2018) 115:1202–10. 10.1016/j.ijbiomac.2018.04.132 29704603

[84] Sun QL Li Y Ni LQ Li YX Cui YS Jiang SL. Structural characterization and antiviral activity of two fucoidans from the brown algae *Sargassum henslowianum*. Carbohydr Polym. (2020) 229:115487. 10.1016/j.carbpol.2019.115487 31826428

[85] Elizondo-Gonzalez R Cruz-Suarez LE Ricque-Marie D Mendoza-Gamboa E Rodriguez-Padilla C Trejo-Avila LM. *In vitro* characterization of the antiviral activity of fucoidan from *Cladosiphon okamuranus* against Newcastle Disease Virus. Virol J. (2012) 9:307. 10.1186/1743-422X-9-307 23234372 PMC3546940

[86] Ponce NMA Flores ML Pujol CA Becerra MB Navarro DA Cordoba O. Fucoidans from the phaeophyta *Scytosiphon lomentaria*: chemical analysis and antiviral activity of the galactofucan component. Carbohydr Res. (2019) 478:18–24. 10.1016/j.carres.2019.04.004 31048118

[87] González ME Alarcón B Carrasco L. Polysaccharides as antiviral agents: antiviral activity of carrageenan. Antimicrob Agents Chemother. (1987) 31:1388–93. 10.1128/AAC.31.9.1388 2823697 PMC174948

[88] Carlucci MJ Pujol CA Ciancia M Noseda MD Matulewicz MC Damonte EB. Antiherpetic and anticoagulant properties of carrageenans from the red seaweed *Gigartina skottsbergii* and their cyclized derivatives: correlation between structure and biological activity. Int J Biol Macromol. (1997) 20:97–105. 10.1016/S0141-8130(96)01145-2 9184941

[89] Girond S Crance JM Van Cuyck-Gandre H Renaudet J Deloince R. Antiviral activity of carrageenan on hepatitis a virus replication in cell culture. Res Virol. (1991) 142:261–70. 10.1016/0923-2516(91)90011-Q 1665574

[90] Diogo JV Novo SG Gonzalez MJ Ciancia M Bratanich AC. Antiviral activity of lambda-carrageenan prepared from red seaweed (*Gigartina skottsbergii*) against BoHV-1 and SuHV-1. Res Vet Sci. (2015) 98:142–4. 10.1016/j.rvsc.2014.11.010 25435342

[91] El-Newary SA Ibrahim AY Asker MS Mahmoud MG El Awady ME. Production, characterization and biological activities of acidic exopolysaccharide from marine *Bacillus amyloliquefaciens* 3MS 2017. Asian Pac J Trop Med. (2017) 10:652–62. 10.1016/j.apjtm.2017.07.005 28870341

[92] Farag MMS Moghannem SAM Shehabeldine AM Azab MS. Antitumor effect of exopolysaccharide produced by *Bacillus mycoides*. Microb Pathog. (2020) 140:103947. 10.1016/j.micpath.2019.103947 31874230

[93] Kumar CG Mongolla P Pombala S. Lasiosan, a new exopolysaccharide from *Lasiodiplodia* sp. strain B2 (MTCC 6000): Structural characterization and biological evaluation. Process Biochem. (2018) 72:162–9. 10.1016/j.procbio.2018.06.014

[94] Lobo RE Gómez MI Font de Valdez G Torino MI. Physicochemical and antioxidant properties of a gastroprotective exopolysaccharide produced by *Streptococcus thermophilus* CRL1190. Food Hydrocoll. (2019) 96:625–33. 10.1016/j.foodhyd.2019.05.036

[95] Sahana TG Rekha PD. A bioactive exopolysaccharide from marine bacteria *Alteromonas* sp. PRIM-28 and its role in cell proliferation and wound healing *in vitro*. Int J Biol Macromol. (2019) 131:10–8. 10.1016/j.ijbiomac.2019.03.048 30851325

[96] Nehal F Sahnoun M Smaoui S Jaouadi B Bejar S Mohammed S. Characterization, high production and antimicrobial activity of exopolysaccharides from *Lactococcus lactis* F-mou. Microb Pathog. (2019) 132:10–9. 10.1016/j.micpath.2019.04.018 31002963

[97] Domingos-Lopes MFP Nagy A Stanton C Ross PR Gelencsér E Silva CCG. Immunomodulatory activity of exopolysaccharide producing *Leuconostoc citreum* strain isolated from Pico cheese. J Funct Foods. (2017) 33:235–43. 10.1016/j.jff.2017.03.054

[98] Mizuno H Tomotsune K Islam MA Funabashi R Albarracin L Ikeda-Ohtsubo W. Exopolysaccharides from *Streptococcus thermophilus* ST538 modulate the antiviral innate immune response in porcine intestinal epithaliocytes. Front Microbiol. (2020) 11:894. 10.3389/fmicb.2020.00894 32508770 PMC7248278

[99] He X Fang J Guo Q Wang M Li Y Meng Y. Advances in antiviral polysaccharides derived from edible and medicinal plants and mushrooms. Carbohydr Polym. (2020) 229:115548. 10.1016/j.carbpol.2019.115548 31826474

[100] Cardozo FT Camelini CM Mascarello A Rossi MJ Nunes RJ Barardi CR. Antiherpetic activity of a sulfated polysaccharide from *Agaricus brasiliensis* mycelia. Antivir Res. (2011) 92:108–14. 10.1016/j.antiviral.2011.07.009 21787804

[101] Rincão VP Yamamoto KA Ricardo NM Soares SA Meirelles LD Nozawa C. Polysaccharide and extracts from *Lentinula edodes*: Structural features and antiviral activity. Virol J. (2012) 9:37. 10.1186/1743-422X-9-37 22336004 PMC3292946

[102] Tochikura TS Nakashima H Hirose K Yamamoto N. A biological response modifier, PSK, inhibits human immunodeficiency virus infection *in vitro*. Biochem Biophys Res Commun. (1987) 2:726–33. 10.1016/0006-291X(87)90936-3 2825669

[103] Tochikura TS Nakashima H Ohashi Y Yamamoto N. Inhibition (*in vitro*) of replication and of the cytopathic effect of human immunodeficiency virus by an extract of the culture medium of *Lentinus edodes* mycelia. Med Microbiol Immunol. (1988) 177:235–44. 10.1007/BF00189409 3173237

[104] Ryu WS. Virus life cycle. In: Ryu WS, editor. Molecular Virology of Human Pathogenic Viruses. Cambridge, MA: Academic Press (2017). p. 31–45.

[105] Seo DJ Changsun C. Antiviral bioactive compounds of mushrooms and their antiviral mechanisms: a review. Viruses. (2021) 13:350. 10.3390/v13020350 33672228 PMC7926341

[106] Wang W Wang SX Guan HS. The antiviral activities and mechanisms of marine polysaccharides: an overview. Mar Drugs. (2012) 10:2795–816. 10.3390/md10122795 23235364 PMC3528127

[107] Shi Q Wang A Lu Z Qin C Hu J Yin J. Overview on the antiviral activities and mechanisms of marine polysaccharides from seaweeds. Carbohydr Res. (2017) 453–454:1–9. 10.1016/j.carres.2017.10.020 29102716

[108] Wang J Wu T Fang X Min W Yang Z. Characterization and immunomodulatory activity of an exopolysaccharide produced by *Lactobacillus plantarum* JLK0142 isolated from fermented dairy tofu. Int J Biol Macromol. (2018) 115:985–93. 10.1016/j.ijbiomac.2018.04.099 29684452

[109] Wang N Jia G Wang C Chen M Xie F Nepovinnykh NV. Structural characterisation and immunomodulatory activity of exopolysaccharides from liquid fermentation of *Monascus purpureus* (Hong Qu). Food Hydrocoll. (2020) 103:105636. 10.1016/j.foodhyd.2019.105636

[110] Liu ZH Niu FJ Xie YX Xie SM Liu YN Yang YY. A review: natural polysaccharides from medicinal plants and microorganisms and their anti-herpetic mechanism. Biomed Pharmacother. (2020) 129:110469. 10.1016/j.biopha.2020.110469 32768956

[111] Liu C Chen H Chen K Gao Y Gao S Liu X. Sulfated modification can enhance antiviral activities of *Achyranthes bidentata* polysaccharide against porcine reproductive and respiratory syndrome virus (PRRSV) *in vitro*. Int J Biol Macromol. (2013) 52:21–4. 10.1016/j.ijbiomac.2012.09.020 23068136

[112] Sinha S Astani A Ghosh T Schnitzler P Ray B. Polysaccharides from *Sargassum tenerrimum*: structural features, chemical modification and anti-viral activity. Phytochemistry. (2010) 71:235–42. 10.1016/j.phytochem.2009.10.014 19931103

[113] Bandyopadhyay SS Navid MH Ghosh T Schnitzler P Ray B. Structural features and *in vitro* antiviral activities of sulfated polysaccharides from *Sphacelaria indica*. Phytochemistry. (2011) 72:276–83. 10.1016/j.phytochem.2010.11.006 21167536

[114] Li S Xiong Q Lai X Li X Wan M Zhang J. Molecular modification of polysaccharides and resulting bioactivities. Compr Rev Food Sci Food Saf. (2016) 15:237–50. 10.1111/1541-4337.12161 33371599

[115] Kalitnik AA Byankina Barabanova AO Nagorskaya VP Reunov AV Glazunov VP Solov'eva TF. Low molecular weight derivatives of different carrageenan types and their antiviral activity. J Appl Phycol. (2012) 25:65–72. 10.1007/s10811-012-9839-8

[116] Mader J Gallo A Schommartz T Handke W Nagel CH Günther P. Calcium spirulan derived from *Spirulina platensis* inhibits herpes simplex virus 1 attachment to human keratinocytes and protects against herpes labialis. J Allergy Clin Immunol. (2016) 137:197–203. 10.1016/j.jaci.2015.07.027 26341274

[117] Kwon PS Oh H Kwon SJ Jin W Zhang F Fraser K. Sulfated polysaccharides effectively inhibit SARS-CoV-2 *in vitro*. Cell Discov. (2020) 6:50. 10.1038/s41421-020-00192-8 32714563 PMC7378085

[118] Lee AJ Ashkar AA. The dual nature of type I and type II interferons. Front Immunol. (2018) 9:2061. 10.3389/fimmu.2018.02061 30254639 PMC6141705

[119] Ivashkiv LB Donlin LT. Regulation of type I interferon responses. Nat Rev Immunol. (2014) 14:36–49. 10.1038/nri3581 24362405 PMC4084561

[120] Hadjadj J Yatim N Barnabei L Corneau A Boussier J Smith N. Impaired type I interferon activity and inflammatory responses in severe COVID-19 patients. Science. (2020) 369:718–24. 10.1126/science.abc6027 32661059 PMC7402632

[121] Hu B Guo H Zhou P Shi ZL. Characteristics of SARS-CoV-2 and COVID-19. Nat Rev Microbiol. (2021) 19:141–54. 10.1038/s41579-020-00459-7 33024307 PMC7537588

[122] Cheng J-J Chao C-H Chang P-C Lu M-K. Studies on anti-inflammatory activity of sulfated polysaccharides from cultivated fungi *Antrodia cinnamomea*. Food Hydrocoll. (2016) 53:37–45. 10.1016/j.foodhyd.2014.09.035

[123] Wu G-J Shiu S-M Hsieh M-C Tsai G-J. Anti-inflammatory activity of a sulfated polysaccharide from the brown alga *Sargassum cristaefolium*. Food Hydrocoll. (2016) 53:16–23. 10.1016/j.foodhyd.2015.01.019

[124] Dinić M Pecikoza U Djokić J Stepanović-Petrović R Milenković M Stevanović M. Exopolysaccharide produced by probiotic strain *Lactobacillus paraplantarum* BGCG11 reduces inflammatory hyperalgesia in rats. Front Pharmacol. (2018) 9:1. 10.3389/fphar.2018.00001 29387012 PMC5776101

[125] Kansandee W Moonmangmee D Moonmangmee S Itsaranuwat P. Characterization and *Bifidobacterium* sp. growth stimulation of exopolysaccharide produced by *Enterococcus faecalis* EJRM152 isolated from human breast milk. Carbohydr Polym. (2019) 206:102–9. 10.1016/j.carbpol.2018.10.117 30553302

[126] Gotteland M Riveros K Gasaly N Carcamo C Magne F Liabeuf G. The pros and cons of using algal polysaccharides as prebiotics. Front Nutr. (2020) 7:163. 10.3389/fnut.2020.00163 33072794 PMC7536576

